# Udca Dosage, Duration, and Initiation Timing for Post-metabolic Bariatric Surgery Scholelithiasis Prevention: A Scoping Review

**DOI:** 10.1007/s11695-026-08544-1

**Published:** 2026-02-27

**Authors:** Matheus Querino da Silva, Mikaell Alexandre Gouvea Faria, João Daniel de Souza Menezes

**Affiliations:** Faculdade de Medicina de São José do Rio Preto, São José do Rio Preto, Brazil

**Keywords:** Severe obesity, Metabolic bariatric surgery, Cholelithiasis, Ursodeoxycholic acid, Prophylaxis

## Abstract

Metabolic Bariatric Surgery (MBS), an effective intervention for severe obesity, elevates the risk of cholelithiasis (CL) due to rapid weight loss and altered biliary metabolism. Ursodeoxycholic acid (UDCA) is increasingly investigated as a prophylactic strategy to mitigate this complication. This scoping review aimed to map and compare UDCA dosage, duration, and initiation timing protocols for CL prevention after MBS. A review was conducted across PubMed/MEDLINE, Web of Science, and Scopus, selecting 10 articles published between 2014 and 2023. The analyzed literature consistently indicates that UDCA prophylaxis significantly reduced CL incidence from 25 to 40% in control groups to 1.4–10.8% in treated groups. Studies commonly reported dosages of 500–600 mg/day for 6 months postoperatively were common, with early initiation being crucial. Efficacy is evident for both Sleeve Gastrectomy (SG) and Roux-en-Y Gastric Bypass (RYGB), with CL relative risks between 0.13 and 0.36. The evidence demonstrates UDCA safety, efficacy, and cost-effectiveness. Collectively, the literature supports UDCA as an effective and safe prophylactic measure against post-MBS cholelithiasis. The identified heterogeneity in protocols underscores the need for standardization to optimize clinical application.

**Supplementary Information:**

The online version contains supplementary material available at 10.1007/s11695-026-08544-1.

## Key Points

The literature consistently indicates UDCA significantly reduces cholelithiasis risk after Metabolic Bariatric Surgery.

Commonly reported UDCA regimens in the literature are 500–600 mg/day for 6 months postoperatively.

Evidence highlights that early initiation of UDCA prophylaxis is critical for optimal outcomes.

Studies demonstrate UDCA use to be safe, effective, and cost-effective.

**Supplementary Information:**

The online version contains supplementary material available at 10.1007/s11695-026-08544-1.

## Introduction

Obesity represents a global public health challenge, with its prevalence on the rise and associated with a complex network of comorbidities that significantly impact individuals’ quality of life and life expectancy [1, 2]. In this scenario, Metabolic Bariatric Surgery (MBS) has emerged as the most effective and durable intervention for promoting substantial weight loss and the resolution or improvement of obesity-related diseases, such as type 2 diabetes and hypertension. However, the optimal clinical outcome of MBS is not without its challenges and postoperative complications [2–8] .

Among the most frequent and clinically relevant complications is the formation of gallstones, known as cholelithiasis. The rapid and intense weight loss induced by MBS profoundly alters biliary metabolism, resulting in cholesterol supersaturation in bile and gallbladder dysmotility, crucial factors for gallstone formation [4]. Post-MBS cholelithiasis can lead to acute cholecystitis, cholangitis, and biliary pancreatitis, requiring medical interventions and, frequently, cholecystectomy. These complications not only compromise patient recovery but also impose a substantial economic burden on healthcare systems due to hospitalizations, additional procedures, and associated treatment costs [8, 9] .

In this context, ursodeoxycholic acid (UDCA), a hydrophilic secondary bile acid, has been widely investigated as a prophylactic strategy to mitigate the risk of cholelithiasis after Metabolic Bariatric Surgery [4]. Its mechanism of action in preventing post-MBS cholelithiasis primarily involves its ability to reduce cholesterol saturation in bile, promote cholesterol solubilization through liquid crystal formation, and improve gallbladder motility [1, 8, 10, 11]. By acting on these pathophysiological mechanisms, UDCA aims to prevent the formation of new gallstones, minimizing complications and the need for additional surgeries, thus becoming an intervention of increasing importance in clinical practice [8, 9, 12].

Considering the continuous increase in the number of MBS procedures performed globally and the inherent risk of cholelithiasis associated with them, as well as the proven efficacy of UDCA as a preventive measure, it becomes essential to understand and optimize the strategies for its application. Given the substantial heterogeneity across study designs, types of metabolic bariatric surgery, ursodeoxycholic acid dosing regimens, treatment durations, initiation timing, and outcome definitions, a scoping review methodology was considered the most appropriate approach to comprehensively map and synthesize the available evidence. This scoping review aimed to map, describe, and compare the ursodeoxycholic acid dosing, duration, and initiation timing protocols reported in the literature for the prevention of cholelithiasis following different types of metabolic bariatric surgery.

## Materials and methods

### Study Design

This scoping review was conducted following the methodological guidelines of the Joanna Briggs Institute (JBI) for scoping reviews and the PRISMA Extension for Scoping Reviews (PRISMA-ScR) reporting items. A scoping review was selected due to the broad nature of the research question and the expected variability in study designs, clinical protocols, and reported outcomes, in accordance with the methodological guidance of the Joanna Briggs Institute and the PRISMA-ScR statement. The protocol followed a five-stage structure: (1) identification of the research question; (2) identification of relevant studies; (3) selection of studies; (4) data extraction; and (5) synthesis and presentation of results [13]. 

## Ethical Aspects

This scoping review exclusively utilized data from previously published and publicly available studies, not directly involving human participants. Therefore, it did not require approval from a research ethics committee. All included studies had their sources properly cited and credited according to academic standards. This article does not contain any studies with human participants or animals performed by any of the authors. For this type of study, formal consent is not required.

## Guiding Question and PICO Strategy

Guiding question: “What are the ursodeoxycholic acid dosage, duration, and initiation timing protocols used for cholelithiasis prevention after different types of Metabolic Bariatric Surgery?” PICO Strategy [13]:

- *P (Population)*: Adult patients (≥ 18 years) undergoing Metabolic Bariatric Surgery.
- *I (Intervention)*: Prophylactic ursodeoxycholic acid.
- *C (Comparison)*: Not applicable (scoping review focused on protocols).
- *O (Outcomes)*: Dosage protocols, treatment duration, initiation timing of therapy, cholelithiasis prevention.

## Protocol Registration

The protocol for this scoping review was prospectively registered in the Open Science Framework (OSF) before data collection began, ensuring methodological transparency and study reproducibility.

## Search Strategy

The search was conducted in the following electronic databases: PubMed/MEDLINE, Web of Science, and Scopus. The search strategy was developed with the assistance of a specialized librarian and adapted for each database, using controlled vocabulary and relevant keywords.

**Table:** 

Database	Search Strategy
PubMed/MEDLINE	(((“Ursodeoxycholic Acid“[Mesh] OR “ursodeoxycholic acid“[tiab] OR “UDCA“[tiab] OR “ursodiol“[tiab]) AND (“Metabolic Bariatric Surgery“[Mesh] OR “Metabolic bariatric surgery“[tiab] OR “gastric bypass“[tiab] OR “sleeve gastrectomy“[tiab] OR “weight loss surgery“[tiab] OR “metabolic surgery“[tiab]) AND (“Cholelithiasis“[Mesh] OR “cholelithiasis“[tiab] OR “gallstones“[tiab] OR “gallstone“[tiab] OR “biliary stones“[tiab] OR “cholecystolithiasis“[tiab]) AND (“prevention“[tiab] OR “prophylaxis“[tiab] OR “preventive“[tiab])))
Web of Science	TS=((“ursodeoxycholic acid” OR “UDCA” OR “ursodiol”) AND (“Metabolic bariatric surgery” OR “gastric bypass” OR “sleeve gastrectomy” OR “weight loss surgery” OR “metabolic surgery”) AND (“cholelithiasis” OR “gallstones” OR “gallstone” OR “biliary stones” OR “cholecystolithiasis”) AND (“prevention” OR “prophylaxis” OR “preventive”))
Scopus	TITLE-ABS-KEY((“ursodeoxycholic acid” OR “UDCA” OR “ursodiol”) AND (“Metabolic bariatric surgery” OR “gastric bypass” OR “sleeve gastrectomy” OR “weight loss surgery” OR “metabolic surgery”) AND (“cholelithiasis” OR “gallstones” OR “gallstone” OR “biliary stones” OR “cholecystolithiasis”) AND (“prevention” OR “prophylaxis” OR “preventive”))

### Eligibility Criteria

Table 1 provides the inclusion and exclusion criteria used for this review.

**Table 1:** Inclusion and exclusion criteria used for this review

Inclusion Criteria	Exclusion Criteria
Population: Adult patients (≥ 18 years) undergoing Metabolic Bariatric Surgery (gastric bypass, sleeve gastrectomy, gastric band, duodenal switch or other bariatric procedures)	**Population**: Pediatric patients (< 18 years); Patients with pre-existing cholelithiasis before MBS procedure; Patients who underwent prior cholecystectomy
Intervention: Use of ursodeoxycholic acid (UDCA) for prophylactic purposes to prevent cholelithiasis Studies reporting at least one of the following: dosage, treatment duration, or initiation timing of therapy	**Intervention**: Use of UDCA exclusively for treating established cholelithiasis Use of other prophylactic agents besides UDCA Studies not specifying UDCA usage protocols
Design: Randomized clinical trials Non-randomized clinical trials Prospective or retrospective cohort studies Case-control studies Systematic reviews with or without meta-analysis Cross-sectional studies reporting protocols	**Design**: Case reports Case series with fewer than 10 patients Letters to the editor Editorials and commentaries Conference abstracts Narrative reviews without systematic methodology
Outcomes: Studies reporting specific UDCA protocols (dosage, duration, initiation timing) Studies that evaluate preventive efficacy of cholelithiasis Studies that report adherence to the prophylactic protocol	**Outcomes**: Studies that evaluate exclusively other outcomes not related to cholelithiasis prevention Studies that do not report data on UDCA protocols
Other criteria: No language restriction No publication date restriction No geographical location restriction Peer-reviewed journal publications	**Other criteria**: Animal studies In vitro studies Unpublished theses and dissertations Duplicates

## Study Selection

Study selection was performed in two stages by three independent reviewers (Reviewer 1, Reviewer 2, Reviewer 3). In the first stage, title and abstract screening was conducted. In the second stage, full-text reading of potentially eligible articles was performed. Discrepancies between the first two reviewers were resolved by consensus or by consulting the third reviewer. The selection process was documented using Rayyan QCRI software to ensure transparency and reproducibility [13]. 

## Data Extraction Instrument

A standardized data extraction instrument was developed and pre-tested on a sample of studies. Extraction was performed independently by two reviewers, with discrepancies resolved by consensus or consultation with the third reviewer. Table 2 outlines the data extraction instrument used for the review.

**Table 2:** Data extraction instrument used for the review

Domain	Extraction Questions
A. STUDY IDENTIFICATION	A1. First author and year of publication A2. Study title A3. Publishing journal A4. Country/region where conducted A5. Language of publication A6. Funding source
B. METHODOLOGICAL CHARACTERISTICS	B1. What was the study design? B2. What was the data collection period? B3. What was the follow-up duration? B4. Was the study multicentric? B5. What was the study setting?
C. STUDY POPULATION	C1. What was the total sample size? C2. How many patients received UDCA? C3. What was the mean/median age of patients? C4. What was the percentage of female patients? C5. What was the mean/median preoperative BMI? C6. What types of **Metabolic Bariatric Surgery** were included?
D. UDCA DOSING PROTOCOL	D1. What UDCA dosage was used? D2. Was the dosage based on body weight? D3. If so, what was the dosage per kg? D4. What was the daily administration frequency? D5. Was the dosage adjusted during treatment? D6. If so, what were the criteria for adjustment?
E. TREATMENT DURATION PROTOCOL	E1. What was the total duration of UDCA treatment? E2. Was the duration predefined or criteria-based? E3. If criteria-based, what were they? E4. Did any patients discontinue earlier than planned? E5. If so, what were the reasons for discontinuation? E6. What was the protocol adherence rate?
F. INITIATION TIMING PROTOCOL	F1. When was UDCA initiated in relation to surgery? F2. If preoperatively, how many days before surgery? F3. If postoperatively, how many days after surgery? F4. Was the initiation timing standardized for all patients? F5. Were there specific criteria for determining initiation? F6. If so, what criteria were used?
G. MONITORING AND FOLLOW-UP	G1. Was monitoring performed during UDCA use? G2. If so, what was the monitoring frequency? G3. What tests were used for monitoring? G4. How was gallstone formation assessed? G5. What was the frequency of assessment for cholelithiasis? G6. Were there predefined criteria to discontinue UDCA?
H. EFFICACY OUTCOMES	H1. What was the incidence of cholelithiasis in the UDCA group? H2. What was the incidence of cholelithiasis in the control group (if applicable)? H3. What was the mean time to cholelithiasis development? H4. Was there a statistically significant difference? H5. What was the calculated preventive efficacy? H6. How many patients required cholecystectomy?
I. SAFETY AND ADVERSE EVENTS	I1. Were UDCA-related adverse events reported? I2. If so, what were the main adverse events? I3. What was the incidence of adverse events? I4. Was there discontinuation due to adverse events? I5. If so, what was the discontinuation rate? I6. Were contraindications to use reported?
J. VARIATIONS BY SURGERY TYPE	J1. Did the protocol vary according to the type of **Metabolic Bariatric Surgery**? J2. If so, what were the differences by surgery type? J3. Was there justification for protocol variations? J4. Which surgery type showed the greatest benefit? J5. Were specific recommendations reported per procedure?
K. LIMITATIONS AND QUALITY	K1. What limitations were reported by the authors? K2. Were conflicts of interest declared? K3. What was the loss to follow-up rate? K4. Did the study have adequate statistical power? K5. Were appropriate randomization methods used (if RCT)? K6. Was adequate blinding used (if applicable)?

### Quality Assessment and Risk of Bias

Although formal quality appraisal is not mandatory for scoping reviews, a methodological quality assessment was conducted to contextualize the heterogeneity of the included studies and to support a critical interpretation of the findings, without excluding studies based on quality criteria.

Methodological quality assessment was performed independently by two reviewers, using appropriate tools for each study type:

- Randomized clinical trials: ROB 2 (Risk of Bias tool version 2) as per Cochrane Handbook 6th edition recommendations.
- Non-randomized intervention studies: ROBINS-I (Risk Of Bias In Non-randomized Studies of Interventions).
- Observational studies: Newcastle-Ottawa Scale or JBI Critical Appraisal Tools.
- Systematic reviews: AMSTAR 2 (A MeaSurement Tool to Assess systematic Reviews) Risk of bias assessment was conducted per outcome or group of outcomes with similar characteristics, with detailed written justification for each judgment domain, as per Cochrane and GRADE recommendations.

### Data Synthesis

Extracted data were synthesized narratively and presented in descriptive tables. Due to the nature of a scoping review, quantitative meta-analysis was not planned. UDCA protocols were categorized and analyzed considering different types of Metabolic Bariatric Surgery, patient characteristics, and clinical contexts. Data were synthesized narratively and descriptively, focusing on mapping patterns, similarities, and variations in ursodeoxycholic acid protocols across studies, rather than on estimating pooled effect sizes.

## Results

### Study Selection Process

The initial search across PubMed/MEDLINE, Web of Science, and Scopus databases led to the identification of 638 articles. After removing 186 duplicates, a total of 452 articles were subjected to title and abstract screening. At this stage, 428 articles were excluded for not meeting eligibility criteria. The remaining 24 articles underwent full-text review. Of these, 14 were excluded for not fitting predefined criteria (e.g., focusing on treatment rather than prevention, or being study protocols), resulting in 10 articles selected for inclusion in this scoping review.

### Descriptive Characteristics of Included Studies

The 10 articles selected for this review were published between 2014 and 2023, with the majority (60%) appearing in the last five years (2019–2023), indicating growing and current interest in preventing post-MBS cholelithiasis with ursodeoxycholic acid (UDCA). In terms of study design, the included studies presented a combination of methodological approaches. We included two Randomized Clinical Trials (RCTs) [10, 14] and one Non-Randomized Clinical Trial (NRCT) [15], representing 30% of the sample. Observational studies comprised three cohort studies, two retrospective [3, 16] and one prospective [17], accounting for another 30%. Additionally, three Systematic Reviews/Meta-analyses [11, 18, 19] and one economic evaluation based on an RCT [20] were included, making up the remaining 40%. The details of these studies are presented in Supplementary Table S1.

The geographical origin of the primary studies reveals contributions from various countries, including France, Egypt, Turkey, Brazil, and Iran, with an economic evaluation based on a Dutch RCT. Meta-analyses naturally encompassed data from multiple global locations.

The sample sizes of primary studies varied significantly, ranging from *N* = 108 [3] to *N* = 2629 [17]. Meta-analyses, by their nature, synthesized data from even larger populations, reaching up to *N* = 2767 [19]. Most studies predominantly focused on patients of the female sex, with percentages ranging from 60% to nearly 80%. The mean age of participants generally fell within the 30–40 year range, and the mean preoperative BMI was consistently above 40 kg/m², reflecting the Metabolic Bariatric Surgery population.

Regarding the types of Metabolic Bariatric Surgery addressed, Sleeve Gastrectomy (SG) and Roux-en-Y Gastric Bypass (RYGB) were the most frequently studied procedures, either individually or in combination. Some studies also included Adjustable Gastric Banding and One-Anastomosis Gastric Bypass.

Concerning UDCA protocols, the most common dosage ranged between 300 mg/day and 600 mg/day, although some studies explored higher doses, such as 900 mg/day or 1200 mg/day. The frequency of administration was generally once or twice daily. The most frequently employed treatment duration was 6 months, with some studies extending up to 12 months. The initiation of UDCA prophylaxis was consistently in the postoperative period, varying from “immediately after” to “30 days after” the surgery. Monitoring of gallstone formation was predominantly performed by abdominal ultrasonography, with frequencies that ranged from quarterly to annually during the follow-up period. The specific details for each study’s protocol can be seen in Supplementary Tables S2 through S11.

Regarding efficacy outcomes, primary studies and meta-analyses consistently demonstrated that UDCA prophylaxis significantly reduces the incidence of cholelithiasis in post-MBS patients. Incidence rates in the UDCA group ranged from 1.4% to 10.8%, in contrast to rates of 25.5% to 40% in untreated control or placebo groups. In addition to reducing gallstone formation, meta-analyses and the economic evaluation study highlighted a reduction in the incidence of symptomatic cholelithiasis and the need for cholecystectomy. The safety profile of UDCA was generally favorable, with reported adverse effects being mild and rare, predominantly gastrointestinal, and low discontinuation rates.

The findings presented reflect reported outcomes from the included studies and should be interpreted as a descriptive synthesis of the existing literature rather than as independent estimates of treatment effectiveness.

## Discussion

This scoping review provides a comprehensive overview of how ursodeoxycholic acid has been utilized in clinical practice for cholelithiasis prevention after metabolic bariatric surgery, highlighting patterns, inconsistencies, and knowledge gaps across heterogeneous study designs.The analysis of the 10 articles selected for this scoping review reinforces the crucial role of ursodeoxycholic acid (UDCA) in preventing cholelithiasis (CL) after Metabolic Bariatric Surgery (MBS). While a consensus on efficacy is evident, the heterogeneity in dosage protocols, duration, and initiation timing of treatment, as well as in the types of MBS, highlights the complexity in optimizing clinical practice.

### UDCA Dosage

Efficacy versus Adherence The daily UDCA dosage employed in primary studies and reviewed in meta-analyses showed a considerable range, but with a concentration around 500–600 mg/day. Studies such as Nabil et al. (2019) [14], Vural et al. (2020) [3], and Salman et al. (2022) [10] used 500 mg/day, while Barzin et al. (2022) opted for 600 mg/day [17]. Machado et al. (2019) investigated 300 mg/day [15]. More detailed information on these protocols can be found in Supplementary Tables S1, S2, S3, S5, S6, S9, S10.

An interesting point of comparison emerged in the study by Coupaye et al. (2017) (Supplementary Table S2), which explored 500 mg/day in a single dose (QD - once daily) versus 250 mg twice daily (BID - twice daily) (totaling 500 mg/day) for Roux-en-Y Gastric Bypass (RYGB) [16]. They observed that 250 mg BID resulted in a CL incidence of 5.7%, significantly lower than 18.6% with 500 mg QD for RYGB (*P* < 0.001). For Sleeve Gastrectomy (SG), the 500 mg QD dose was highly effective, with a CL incidence of only 2.4% (*P* = 0.005). It is important to note that this study is retrospective and non-randomized, which may introduce selection bias and limit the comparability of the groups. This data suggests that, especially for RYGB, the frequency of administration, rather than the total daily dose, may influence efficacy, possibly due to optimized absorption or maintenance of plasma levels.

The meta-analysis by Fearon et al. (2022) (Supplementary Table S4) complements this discussion by indicating that dosages ≤ 600 mg/day are associated with better adherence and clinical outcomes [18]. They demonstrated that higher doses, such as 1000 mg or 1200 mg, which were used in some primary studies, were associated with a higher rate of adverse events and lower adherence. This reinforces that the dose-response relationship may not be linear and that the “ideal dose” must balance efficacy and tolerability.

### Treatment Duration

Covering the Risk Window Most primary studies adopted a 6-month duration for UDCA prophylaxis (Nabil et al., 2019 [14]; Coupaye et al., 2017 [16]; Barzin et al., 2022 [17]; Haal et al., 2022 [20]). Machado et al. (2019) [15] used 5 months. However, Salman et al. (2022) [10] extended treatment to 12 months. This decision is based on the understanding that the peak incidence of symptomatic cholelithiasis can occur over a longer period, reported up to 16 months after surgery. The meta-analysis by Stokes et al. (2014) [11], although not delving into specific durations, contextualizes that the first 6 months represent the period of greatest weight loss and, consequently, of highest metabolic risk for biliary lithogenesis.

### Initiation Timing

The Urgency of Early Intervention The most striking consistency among studies lies in initiating prophylaxis in the postoperative period, with no evidence of preoperative use. The meta-analysis by Stokes et al. (2014) [11] emphasizes the importance of initiation “immediately after” the surgery, citing that gallstone formation can begin as early as the first 4 weeks of accelerated weight loss.

Most studies do not detail an exact number of postoperative days for initiation, generically referring to a “postoperative” regimen. Machado et al. (2019) [15] specified initiation 30 days after the surgery. It should be considered, however, the limitations of this study, such as its non-randomized design and lack of blinding, which may impact the rigorous interpretation of optimal timing. Considering the rapid change in bile composition and gallbladder motility induced by weight loss, an earlier initiation is theoretically more advantageous.

Efficacy by Metabolic Bariatric Surgery Type Studies primarily covered SG and RYGB, the most common MBS procedures. UDCA efficacy was demonstrated in both.

- SG/LSG (Laparoscopic Sleeve Gastrectomy): Nabil et al. (2019) [14] demonstrated significant reductions in CL after LSG from 40% in control to 6% in the UDCA group (*p* < 0.001). Salman et al. (2022) ([10] reported a reduction from 32% to 8.5% (*p* < 0.001). It is important to consider that the study by Salman et al. (2022) had limitations such as lack of blinding, non-standardized postoperative dietary habits, and a loss to follow-up of 21.4% of patients. Vural et al. (2020) [3] found a drop from 33.33% to 10.18% (*p* = 0.007). Vural et al. (2020) was a retrospective cohort study, which inherently makes it susceptible to selection and confounding biases [3].
- RYGB: Machado et al. (2019) [15] observed a drastic reduction from 26.4% in control to 1.4% in the UDCA group (Odds Ratio [OR] = 24.4, *P* < 0.001). However, the interpretation of this result must consider the non-randomized design and the absence of blinding in the study, which introduce the risk of selection and observation bias.

The meta-analysis by Fearon et al. (2022) [18] provided a comparative overview: for SG, UDCA resulted in 11.8% CL incidence versus 37% in controls (Relative Risk [RR] 0.32); for RYGB, 8.6% versus 25.7% (RR 0.37). This indicates efficacy in both, with similar RRs. However, meta-analyses also highlight the significant heterogeneity among primary studies and the high dropout rates in some of them, which may affect the robustness of pooled estimates. Barzin et al. (2022) [17], in their massive prospective cohort (*N* = 2629) under UDCA prophylaxis, observed that the procedure type (SG vs. GB) was not an independent predictor of cholelithiasis at 24 months.

### Overall Efficacy and Cost-effectiveness Meta-analyses Consolidated the Robust Efficacy of UDCA

- Fearon et al. (2022) [18]: Overall RR of 0.36 (95% Confidence Interval [CI] 0.24–0.51, *p* < 0.00001) for CL formation, with a Number Needed to Treat (NNT) of 9. Reduction in symptomatic CL was RR 0.24 (*p* < 0.00001).
- Al-huniti et al. (2023) [19]: Overall RR of 0.13 (*p* < 0.0001) for CL. Reduction in symptomatic CL (RR 5.70 for control, *p* < 0.00001) and cholecystectomy (RR 3.05 for control, *p* = 0.002).
- Stokes et al. (2014) [11]: Overall RR of 0.33 (95% CI 0.18–0.60) for CL, NNT = 9. Reduction in cholecystectomy (RR 0.20, NNT = 15). While meta-analyses provide strong evidence, it is important to acknowledge that the heterogeneity among primary studies in terms of design, populations, and UDCA regimens is a limitation pointed out by the authors themselves, requiring caution in the unrestricted generalization of results.

The economic evaluation by Haal et al. (2022) [20] further strengthens the argument, demonstrating that UDCA prophylaxis (900 mg/day for 6 months) in RYGB patients without pre-existing gallstones is cost-effective. The UDCA group had 96.6% of patients free of symptomatic disease at 24 months, versus 90.8% in placebo (relative risk 1.06, *p* = 0.002 for “remaining free”). This resulted in an average gain of 0.047 QALYs (Quality-Adjusted Life Years) (*p* = 0.022) and non-significant cost savings of -€1392 per patient from a societal perspective, with an 87.2% probability of cost-effectiveness. The limitations of this study include the need for data imputation and the non-collection of information on certain costs and travel expenses, in addition to a 2-year time horizon, which may not capture all lifetime costs and benefits.

Safety, Adherence, and Predictors UDCA demonstrated a favorable safety profile. Adverse events were predominantly mild and gastrointestinal, with low discontinuation rates. Nabil et al. (2019) [14] reported only 3 patients with mild nausea/vomiting. Salman et al. (2022) [10] observed that 3 patients (2.2%) discontinued due to severe adverse events. Barzin et al. (2022) [17] reported 15 patients (0.57%) with rashes that led to discontinuation. These rates are generally low, contributing to the viability of prophylaxis.

Identifying predictors of cholelithiasis, even under UDCA prophylaxis, is crucial for refining patient selection. Barzin et al. (2022) [17] identified BMI (Body Mass Index) loss at 6 months as the only independent and significant predictor of cholelithiasis at 24 months (Hazard Ratio [HR] = 1.10 [95% CI: 1.04–1.16], *p* = 0.001). At 12 months, both preoperative BMI (HR = 1.03 [1.00–1.05], *p* = 0.04) and BMI loss at 6 months (HR = 1.08 [1.02–1.14], *p* = 0.02) were predictors. It is relevant to note that this study by Barzin et al. (2022) was a retrospective analysis of prospective data, and ultrasonographic evaluation of the gallbladder in patients with obesity can be inconsistent, which represents a limitation. Salman et al. (2022) [10] also highlighted higher BMI, dyslipidemia, and absence of prophylaxis as independent factors, even for patients taking UDCA.

Across the included studies, ursodeoxycholic acid use was consistently associated with lower reported rates of postoperative cholelithiasis when compared with control or placebo groups, although the magnitude of this association varied substantially depending on study design and protocol characteristics.

### Gaps and Future Directions

As a scoping review, this study did not aim to formally synthesize effect sizes or establish causal relationships. Additionally, the inclusion of heterogeneous study designs and outcome definitions limits direct comparability across studies.

Despite strong evidence of efficacy and cost-effectiveness, heterogeneity in UDCA protocols and the complex interaction between surgery type, dosage, and administration frequency still represent challenges. The variability in study populations, geographical regions, and clinical settings among primary studies may limit the generalizability of specific findings. Future studies should focus on more standardized Randomized Clinical Trials (RCTs), with prolonged follow-up to assess long-term outcomes. The investigation of biomarkers that can predict the individual response to UDCA or the risk of gallstone formation, allowing for personalized prophylaxis, is a promising direction.

## Conclusion

This scoping review mapped the existing evidence on ursodeoxycholic acid prophylaxis after metabolic bariatric surgery, demonstrating considerable variability in dosing, duration, and initiation timing protocols. The studies consistently indicate a significant reduction in the incidence of CL, both asymptomatic and symptomatic, and in the need for cholecystectomy. UDCA prophylaxis also proves to be cost-effective, offering clinical and economic benefits.

Despite proven efficacy, there is heterogeneity in UDCA dosage, duration, and initiation timing protocols among the studies. The most common dosage varies between 500 and 600 mg/day, with a predominant duration of 6 months, initiated postoperatively. Rapid weight loss emerges as a relevant predictor.

Further research focused on standardizing protocols adapted to surgery types and patient characteristics is recommended, aiming to optimize clinical practice.

While the literature consistently suggests a protective association against cholelithiasis, standardized protocols and high-quality randomized trials are needed to optimize clinical practice.

## Supplementary Information

Below is the link to the electronic supplementary material.

Supplementary Material 1 (DOCX 162 KB)

## Data Availability

No datasets were generated or analysed during the current study.
