# Supplementary material for: Udca Dosage, Duration, and Initiation Timing for Post-metabolic Bariatric Surgery Scholelithiasis Prevention: A Scoping Review
Source: Obes Surg. 2026 Feb 27;36(4):1890–8. doi: 10.1007/s11695-026-08544-1 (PMC13083534; doi:10.1007/s11695-026-08544-1)
Supplement: Supplementary file 1 — Supplementary Material 1 (DOCX 162 KB) [file 11695_2026_8544_MOESM1_ESM.docx]

Supplemental Material

Table S1. Details of the articles included in the review.

| ID | Title | Author, Year | Country of Origin | Design | Population (N, Surgery, Key Data) | Main Results (UDCA Protoc., Efficacy, Safety) | Conclusion |
| --- | --- | --- | --- | --- | --- | --- | --- |
| 1 | Evaluation of incidence of cholelithiasis after bariatric surgery in subjects treated or not treated with ursodeoxycholic acid | Coupaye et al., 2017 | France | Retrospective Cohort Study | N=399 (231 UDCA, 168 untreated); SG and RYGB. Mean age ~42 years, ~85% female. | UDCA 500mg once daily effective for SG (reduction from 25.5% to 2.4% cholelithiasis). Twice daily doses (250mg twice daily) more effective for RYGB (reduction from 32.5% to 5.7% cholelithiasis) than 500mg once daily (18.6% cholelithiasis). Treatment for 6 months postoperatively. High adherence. | UDCA 500mg once daily for 6 months is effective in preventing cholelithiasis 1 year after SG, but twice daily doses appear more effective after RYGB. |
| 2 | Effect of oral ursodeoxycholic acid on cholelithiasis following laparoscopic sleeve gastrectomy for morbid obesity | Nabil et al., 2019 | Egypt | Randomized Clinical Trial | N=200 (100 UDCA, 100 Control); LSG. Mean age ~31 years, ~77% female. | UDCA 250mg twice daily (500mg/day) for 6 months postoperatively. Reduced incidence of cholelithiasis from 40% (control) to 6% (UDCA). High adherence (100%). Mild adverse events (nausea/vomiting in 3 patients). | UDCA treatment for 6 months after LSG is effective in preventing cholelithiasis. |
| 3 | The impact of ursodeoxycholic acid on gallstone disease after bariatric surgery: a meta-analysis of randomized control trials | Fearon et al., 2022 | Global (Meta-analysis) | Systematic Review and Meta-analysis (10 RCTs) | N=2583 patients (1772 UDCA, 811 Placebo); Various types of bariatric surgery. Mean age ~30-46 years, ~66% female. | UDCA (RR 0.36; P < 0.00001) significantly reduced gallstone formation in 7.3% vs 24.7% (control). Dosage ≤600mg/day more effective. Variable duration (generally 6 months). Initiated postoperatively. Reduced symptomatic gallstones and need for cholecystectomy. Mild AEs, ranging from 4.8% to 20.9%. | UDCA significantly reduces the risk of asymptomatic and symptomatic gallstones after bariatric surgery. A dose of 600 mg/day or less appears more effective and with better adherence. |
| 4 | Increased gallstone formation after sleeve gastrectomy and the preventive role of ursodeoxycholic acid | Vural et al., 2020 | Turkey | Retrospective Cohort Study | N=108 (42 UDCA, 66 untreated); SG. Mean age ~40 years, ~67% female. | UDCA 500mg/day (2x250mg) postoperatively (duration not specified, but commonly 6 months). Incidence of cholelithiasis in UDCA group 10.18% vs 33.33% in untreated group. Statistically significant difference (p=0.007). | Prophylactic use of UDCA significantly reduced gallstone formation after weight loss surgery. BMI loss rate was a predictor. |
| 5 | Ursodeoxycholic acid in the prevention of gallstones in patients subjected to Roux-en-Y gastric bypass | Machado et al., 2019 | Brazil | Prospective Comparative Clinical Study (Non-Randomized) | N=137 (69 UDCA, 68 Control); RYGB. Mean age ~35 years, ~67% female. | UDCA 150mg twice daily (300mg/day) for 5 months. Initiated 30 days postoperatively. Reduced incidence of cholelithiasis from 26.4% (control) to 1.4% (UDCA). Adherence 100%. | The efficacy of UDCA in preventing gallstone formation in patients postoperative bariatric surgery is proven. Untreated patients with UDCA were 24.4x more likely to develop cholelithiasis. |
| 6 | Ursodeoxycholic Acid Prophylaxis and the Reduction of Gallstone Formation After Bariatric Surgery: An Updated Meta-Analysis of Randomized Controlled Trials | Al-huniti et al., 2023 | Global (Meta-analysis) | Systematic Review and Meta-analysis (12 RCTs) | N=2767 patients; Various types of bariatric surgery. Baseline age, sex, BMI comparable. | UDCA (RR 0.13; P < 0.0001) reduced the overall incidence of cholelithiasis after bariatric surgery. Reduction observed at 3, 6, and 12 months. Reduced incidence of symptomatic cholelithiasis (RR 5.70; P < 0.00001) and cholecystectomy rate (RR 3.05; P = 0.002). Dosage and duration varied among primary studies. | UDCA prophylaxis is effective in preventing gallstone formation after bariatric surgery, reducing overall and symptomatic incidence, and the need for cholecystectomy. |
| 7 | Ursodeoxycholic Acid and Diets Higher in Fat Prevent Gallbladder Stones During Weight Loss: A Meta-analysis of Randomized Controlled Trials | Stokes et al., 2014 | Global (Meta-analysis) | Systematic Review and Meta-analysis (11 RCTs with UDCA) | N=1836 participants (in 13 RCTs, 11 with UDCA); Obese on diet or post-bariatric surgery. Mean age not consolidated, ~42-100% female. | UDCA reduced the risk of cholelithiasis (RR 0.33; NNT=9). Duration from 6 weeks to 18 months. Dose of 300-1200mg/day (median 750mg/day). Greater effect in diets (RR 0.17) vs bariatric surgery (RR 0.42). Reduced risk of cholecystectomy (RR 0.20; NNT=15). Mild and rare adverse events. | UDCA and/or higher fat diets can prevent gallstone formation during weight loss. |
| 8 | Ursodeoxycholic acid for the prevention of gall stones after laparoscopic sleeve gastrectomy: a prospective controlled study | Salman et al., 2022 | Not reported (Egypt inferred) | Prospective Randomized Clinical Trial | N=258 (130 UDCA, 128 Control); LSG. Mean age ~37 years, ~64% female. | UDCA 500mg once daily for 12 months postoperatively. Reduced incidence of cholelithiasis from 32% (control) to 8.5% (UDCA) in the first year (p < 0.001). Reduced cholecystectomy from 7% to 2.3% (p=0.072). Severe AEs in 3 patients (allergy, GI) led to exclusion. | UDCA 500mg once daily for 12 months after LSG is effective in reducing gallstone formation and the need for cholecystectomy. |
| 9 | Predictive Factors of Cholelithiasis After Prophylactic Administration of Ursodeoxycholic Acid Following Laparoscopic Bariatric Surgery: Tehran Obesity Treatment Study | Barzin et al., 2022 | Iran | Prospective Cohort Study | N=2629 (all received UDCA); SG and GB. Mean age 38.8 years, 79.7% female. | UDCA 300mg twice daily (600mg/day) for 6 months postoperatively. Cumulative incidence of cholelithiasis of 10.8% at 24 months. Higher incidence in the first year. BMI loss at 6 months was the only independent predictor of de novo cholelithiasis. AEs (rashes) in 15 patients (0.57%). | The incidence of cholelithiasis is significantly reduced with UDCA. Rapid postoperative weight loss (BMI at 6 months) is the main predictor of de novo cholelithiasis, albeit with low discriminatory power. |
| 10 | Cost-effectiveness of ursodeoxycholic acid in preventing new-onset symptomatic gallstone disease after Roux-en-Y gastric bypass surgery | Haal et al., 2022 | Netherlands | Economic Evaluation of RCT | N=705 (348 UDCA, 357 Placebo); RYGB (patients without pre-surgical gallstones). Mean age ~44 years, ~76% female. | UDCA 900mg/day for 6 months postoperatively. Reduced symptomatic disease from 9.2% (placebo) to 3.4% (UDCA) (RR 1.06; P = 0.002 for "remaining free"). Resulted in greater QALY gain (0.047) and cost savings (–€1392) from a societal perspective. | UDCA prophylaxis after RYGB in patients without gallstones before surgery was cost-effective for preventing symptomatic gallstone disease. |

Table S2. Data extraction details - Coupaye et al., 2017

| Domain | Extraction Questions | Detailed Answers |
| --- | --- | --- |
| A. STUDY IDENTIFICATION | A1. First author and year of publication | Coupaye et al., 2017 |
|  | A2. Study title | Evaluation of incidence of cholelithiasis after bariatric surgery in subjects treated or not treated with ursodeoxycholic acid |
|  | A3. Publishing journal | Surgery for Obesity and Related Diseases |
|  | A4. Country/region where conducted | France |
|  | A5. Language of publication | English |
|  | A6. Funding source | Not reported (The authors declare no commercial associations that could represent a conflict of interest.) |
| B. METHODOLOGICAL CHARACTERISTICS | B1. What was the study design? | Retrospective cohort study (analysis of prospective database) |
|  | B2. What was the data collection period? | Surgeries performed between January 2008 and February 2015. Followed until April 2016. |
|  | B3. What was the follow-up duration? | Mean of 13.0 ± 3.4 months. |
|  | B4. Was the study multicentric? | No (single university hospital) |
|  | B5. What was the study setting? | University hospital |
| C. STUDY POPULATION | C1. What was the total sample size? | 399 patients included in the analysis. |
|  | C2. How many patients received UDCA? | 231 patients (SG: 42; RYGB 250 mg twice daily: 87; RYGB 500 mg once daily: 102). |
|  | C3. What was the mean/median age of patients? | SG (without UDCA): 43.0 ± 9.7 years; SG (with UDCA): 41.4 ± 11.4 years; RYGB (without UDCA): 41.4 ± 11.5 years; RYGB (with UDCA 250 mg twice daily): 41.0 ± 10.4 years; RYGB (with UDCA 500 mg once daily): 43.1 ± 11.1 years. |
|  | C4. What was the percentage of female patients? | SG (without UDCA): 88.2%; SG (with UDCA): 85.7%; RYGB (without UDCA): 90.6%; RYGB (with UDCA 250 mg twice daily): 85.1%; RYGB (with UDCA 500 mg once daily): 82.4%. |
|  | C5. What was the mean/median preoperative BMI? | SG (without UDCA): 44.8 ± 8.3 kg/m²; SG (with UDCA): 43.9 ± 6.9 kg/m²; RYGB (without UDCA): 44.9 ± 5.0 kg/m²; RYGB (with UDCA 250 mg twice daily): 43.9 ± 5.2 kg/m²; RYGB (with UDCA 500 mg once daily): 44.1 ± 4.7 kg/m². |
|  | C6. What types of bariatric surgery were included? | Sleeve Gastrectomy (SG) and Roux-en-Y Gastric Bypass (RYGB). |
| D. UDCA DOSING PROTOCOL | D1. What UDCA dosage was used? | 500 mg/day (total dose) |
|  | D2. Was the dosage based on body weight? | No. |
|  | D3. If so, what was the dosage per kg? | Not applicable. |
|  | D4. What was the daily administration frequency? | Once daily (500mg) or twice daily (250mg). |
|  | D5. Was the dosage adjusted during treatment? | Not reported as individual adjustment, but there was a change in the dosing protocol over time. |
|  | D6. If so, what were the criteria for adjustment? | Initially 250 mg twice daily for RYGB (Feb/2012), then 500 mg once daily for both procedures (from 2013 onwards), to "improve adherence" and due to the "most common presentation available in France." |
| E. TREATMENT DURATION PROTOCOL | E1. What was the total duration of UDCA treatment? | 6 months. |
|  | E2. Was the duration predefined or criteria-based? | Predefined. |
|  | E3. If criteria-based, what were they? | Not applicable. |
|  | E4. Did any patients discontinue earlier than planned? | Yes (4 patients: 3 in SG group and 1 in RYGB group). |
|  | E5. If so, what were the reasons for discontinuation? | Not related to side effects. Patients with early postoperative complications who could not take UDCA for several weeks were excluded. |
|  | E6. What was the protocol adherence rate? | High ("high compliance observed in our study"), attributed to reimbursement of treatment by the French health system and systematic information to patients about the risk of cholelithiasis. |
| F. INITIATION TIMING PROTOCOL | F1. When was UDCA initiated in relation to surgery? | Postoperatively. |
|  | F2. If preoperatively, how many days before surgery? | Not applicable. |
|  | F3. If postoperatively, how many days after surgery? | Not specified in exact days, but "systematically prescribed... after surgery". |
|  | F4. Was the initiation timing standardized for all patients? | Yes (systematic prescription for patients without prior cholecystectomy and intact gallbladder). |
|  | F5. Were there specific criteria for determining initiation? | No, the decision to systematically prescribe UDCA was based on previous studies by the group. |
|  | F6. If so, what criteria were used? | Implementation from February 2012 for RYGB and October 2013 for SG. |
| G. MONITORING AND FOLLOW-UP | G1. Was monitoring performed during UDCA use? | Yes. |
|  | G2. If so, what was the monitoring frequency? | Scheduled ultrasound at 6 months and 1 year postoperatively. |
|  | G3. What tests were used for monitoring? | Not reported (beyond ultrasound for cholelithiasis). |
|  | G4. How was gallstone formation assessed? | Abdominal ultrasonography. |
|  | G5. What was the frequency of cholelithiasis assessment? | At 6 months and 1 year postoperatively. |
|  | G6. Were there predefined criteria to discontinue UDCA? | Not reported, except for interruption due to early postoperative complications preventing medication intake. |
| H. EFFICACY OUTCOMES | H1. What was the incidence of cholelithiasis in the UDCA group? | SG with UDCA (500 mg once daily): 2.4%; RYGB with UDCA (250 mg twice daily): 5.7%; RYGB with UDCA (500 mg once daily): 18.6%. |
|  | H2. What was the incidence of cholelithiasis in the control group (if applicable)? | SG without UDCA: 25.5%; RYGB without UDCA: 32.5%. |
|  | H3. What was the mean time to cholelithiasis development? | Not specifically reported, but "about 80% of cholelithiasis occurred in the first year." |
|  | H4. Was there a statistically significant difference? | Yes (P < 0.05 to P < 0.001, depending on the group). |
|  | H5. What was the calculated preventive efficacy? | Significant reduction: SG from 25.5% to 2.4%; RYGB (untreated) from 32.5% to 5.7% (250mg BID) and 18.6% (500mg QD). |
|  | H6. How many patients required cholecystectomy? | UDCA reduced the number of cholecystectomies due to symptoms from 22 to 6 after bariatric surgery (5 to 0 after SG and 17 to 6 after RYGB). |
| I. SAFETY AND ADVERSE EVENTS | I1. Were UDCA-related adverse events reported? | Yes, but in a very low number. |
|  | I2. If so, what were the main adverse events? | Not specified. Only that 4 patients discontinued and "were never related to side effects." |
|  | I3. What was the incidence of adverse events? | Low. |
|  | I4. Was there discontinuation due to adverse events? | No (discontinuations were not due to adverse events). |
|  | I5. If so, what was the discontinuation rate? | Not applicable. |
|  | I6. Were contraindications to use reported? | Not explicitly reported, but patients with "early postoperative complications who could not take UDCA for several weeks were excluded." |
| J. VARIATIONS BY SURGERY TYPE | J1. Did the protocol vary according to the type of bariatric surgery? | Yes, initially. |
|  | J2. If so, what were the differences by surgery type? | RYGB: 250 mg twice daily (initiation Feb/2012); SG: 500 mg once daily (initiation Oct/2013). Subsequently, 500 mg once daily for both. |
|  | J3. Was there justification for protocol variations? | To improve adherence (low adherence reported for BID doses) and to correspond with the most common UDCA presentation available in France. |
|  | J4. Which surgery type showed the greatest benefit? | SG with UDCA 500 mg once daily (2.4% cholelithiasis) had a lower incidence than RYGB with 500 mg once daily (18.6%). However, RYGB with 250 mg twice daily (5.7%) was more effective than RYGB with 500 mg once daily. |
|  | J5. Were specific recommendations reported per procedure? | Yes. UDCA 500 mg once daily effective for SG. Twice daily doses appear more effective after RYGB. |
| K. LIMITATIONS AND QUALITY | K1. What limitations were reported by the authors? | 1) Not randomized; 2) treated and untreated groups not followed in simultaneous periods; 3) very short follow-up for long-term conclusions; 4) low number of SG patients initially limited statistical power. |
|  | K2. Were conflicts of interest declared? | No ("The authors have no commercial associations that could be a conflict of interest"). |
|  | K3. What was the loss to follow-up rate? | Not specified for the treated vs untreated group directly, but 7% of patients with ultrasound at 6 months did not undergo ultrasound at 1 year. |
|  | K4. Did the study have adequate statistical power? | Limited for subgroup comparisons with SG due to the low number of patients. |
|  | K5. Were appropriate randomization methods used (if RCT)? | Not applicable (not randomized). |
|  | K6. Was adequate blinding used (if applicable)? | Not applicable (not randomized). |

Table S3. Data extraction details - Nabil et al., 2019

| Domain | Extraction Questions | Detailed Answers |
| --- | --- | --- |
| A. STUDY IDENTIFICATION | A1. First author and year of publication | Nabil et al., 2019 |
|  | A2. Study title | Effect of oral ursodeoxycholic acid on cholelithiasis following laparoscopic sleeve gastrectomy for morbid obesity |
|  | A3. Publishing journal | Surgery for Obesity and Related Diseases |
|  | A4. Country/region where conducted | Egypt |
|  | A5. Language of publication | English |
|  | A6. Funding source | Not reported (The authors declare no commercial associations that could represent a conflict of interest.) |
| B. METHODOLOGICAL CHARACTERISTICS | B1. What was the study design? | Randomized Clinical Trial (RCT) and Prospective |
|  | B2. What was the data collection period? | July 2015 to March 2018. |
|  | B3. What was the follow-up duration? | 1 year. |
|  | B4. Was the study multicentric? | Yes (Two university hospitals in Egypt) |
|  | B5. What was the study setting? | University hospitals |
| C. STUDY POPULATION | C1. What was the total sample size? | 200 patients included in the analysis. |
|  | C2. How many patients received UDCA? | 100 patients. |
|  | C3. What was the mean/median age of patients? | UDCA group: 30.4 ± 5.9 years; Control: 31.7 ± 5.4 years. |
|  | C4. What was the percentage of female patients? | UDCA group: 79%; Control: 75%. |
|  | C5. What was the mean/median preoperative BMI? | UDCA group: 47.1 ± 5.1 kg/m²; Control: 45.8 ± 6.3 kg/m². |
|  | C6. What types of bariatric surgery were included? | Laparoscopic Sleeve Gastrectomy (LSG). |
| D. UDCA DOSING PROTOCOL | D1. What UDCA dosage was used? | 500 mg/day (250 mg x 2). |
|  | D2. Was the dosage based on body weight? | No. |
|  | D3. If so, what was the dosage per kg? | Not applicable. |
|  | D4. What was the daily administration frequency? | Twice daily. |
|  | D5. Was the dosage adjusted during treatment? | Not reported. |
|  | D6. If so, what were the criteria for adjustment? | Not applicable. |
| E. TREATMENT DURATION PROTOCOL | E1. What was the total duration of UDCA treatment? | 6 months. |
|  | E2. Was the duration predefined or criteria-based? | Predefined. |
|  | E3. If criteria-based, what were they? | Not applicable. |
|  | E4. Did any patients discontinue earlier than planned? | No (all patients followed the complete regimen). |
|  | E5. If so, what were the reasons for discontinuation? | Not applicable. |
|  | E6. What was the protocol adherence rate? | 100% (confirmed by phone calls and follow-up visits). |
| F. INITIATION TIMING PROTOCOL | F1. When was UDCA initiated in relation to surgery? | Postoperatively. |
|  | F2. If preoperatively, how many days before surgery? | Not applicable. |
|  | F3. If postoperatively, how many days after surgery? | Not specified in exact days, but "prophylaxis in the form of oral UDCA... for 6 months". |
|  | F4. Was the initiation timing standardized for all patients? | Yes (postoperative prophylaxis regimen). |
|  | F5. Were there specific criteria for determining initiation? | No (systematic prescription). |
|  | F6. If so, what criteria were used? | Not applicable. |
| G. MONITORING AND FOLLOW-UP | G1. Was monitoring performed during UDCA use? | Yes. |
|  | G2. If so, what was the monitoring frequency? | Monthly (outpatient clinic to monitor weight loss and postoperative problems). |
|  | G3. What tests were used for monitoring? | Not reported (beyond ultrasound for cholelithiasis). |
|  | G4. How was gallstone formation assessed? | Abdominal ultrasonography. |
|  | G5. What was the frequency of cholelithiasis assessment? | Every 3 months for 1 year (at 3, 6, 9, and 12 months). |
|  | G6. Were there predefined criteria to discontinue UDCA? | Not reported. |
| H. EFFICACY OUTCOMES | H1. What was the incidence of cholelithiasis in the UDCA group? | 6% (6 out of 100 patients). |
|  | H2. What was the incidence of cholelithiasis in the control group (if applicable)? | 40% (40 out of 100 patients). |
|  | H3. What was the mean time to cholelithiasis development? | Not reported an exact mean time. 65% diagnosed at 6 months, 24% at 9 months, 11% at 12 months. |
|  | H4. Was there a statistically significant difference? | Yes (P < 0.001). |
|  | H5. What was the calculated preventive efficacy? | Significant reduction (from 40% to 6%). |
|  | H6. How many patients required cholecystectomy? | Not specified directly the number for each group, but 31 symptomatic patients (27 control, 4 UDCA) underwent cholecystectomy. |
| I. SAFETY AND ADVERSE EVENTS | I1. Were UDCA-related adverse events reported? | Yes. |
|  | I2. If so, what were the main adverse events? | Nausea and vomiting. |
|  | I3. What was the incidence of adverse events? | Only 3 patients (not bothersome enough to stop medication). |
|  | I4. Was there discontinuation due to adverse events? | No. |
|  | I5. If so, what was the discontinuation rate? | Not applicable. |
|  | I6. Were contraindications to use reported? | Not reported. |
| J. VARIATIONS BY SURGERY TYPE | J1. Did the protocol vary according to the type of bariatric surgery? | No, only LSG was included. |
|  | J2. If so, what were the differences by surgery type? | Not applicable. |
|  | J3. Was there justification for protocol variations? | Not applicable. |
|  | J4. Which surgery type showed the greatest benefit? | Not applicable. |
|  | J5. Were specific recommendations reported per procedure? | Not applicable. |
| K. LIMITATIONS AND QUALITY | K1. What limitations were reported by the authors? | Relatively short follow-up (12 months). |
|  | K2. Were conflicts of interest declared? | No ("The authors have no commercial associations that could be a conflict of interest"). |
|  | K3. What was the loss to follow-up rate? | No explicit loss to follow-up reported, all patients were available for assessment at the end of follow-up. |
|  | K4. Did the study have adequate statistical power? | Not specified in the text. |
|  | K5. Were appropriate randomization methods used (if RCT)? | Yes (random allocation into 1 of 2 groups). |
|  | K6. Was adequate blinding used (if applicable)? | Not reported (inferred that there was no blinding). |

Table S4. Data extraction details - Fearon et al., 2022

| Domain | Extraction Questions | Detailed Answers |
| --- | --- | --- |
| A. STUDY IDENTIFICATION | A1. First author and year of publication | Fearon et al., 2022 |
|  | A2. Study title | The impact of ursodeoxycholic acid on gallstone disease after bariatric surgery: a meta-analysis of randomized control trials |
|  | A3. Publishing journal | Surgery for Obesity and Related Diseases |
|  | A4. Country/region where conducted | Not applicable (Global Meta-analysis) |
|  | A5. Language of publication | English |
|  | A6. Funding source | Not reported (The authors declare no commercial associations that could represent a conflict of interest.) |
| B. METHODOLOGICAL CHARACTERISTICS | B1. What was the study design? | Systematic review and Meta-analysis of Randomized Clinical Trials (RCTs) |
|  | B2. What was the data collection period? | Electronic search until October 2020. Included articles published between 1993 and 2020. |
|  | B3. What was the follow-up duration? | Variable among included studies (Minimum 3 months, Maximum 24 months) |
|  | B4. Was the study multicentric? | Not applicable (Meta-analysis of studies) |
|  | B5. What was the study setting? | Not applicable (Meta-analysis of studies) |
| C. STUDY POPULATION | C1. What was the total sample size? | 2583 patients in the 10 included RCTs. |
|  | C2. How many patients received UDCA? | 1772 patients. |
|  | C3. What was the mean/median age of patients? | Variable between 30.1 and 46 years in studies that reported. |
|  | C4. What was the percentage of female patients? | 66.2% (1058/1686 patients in studies that reported). |
|  | C5. What was the mean/median preoperative BMI? | Variable between 42.7 and 50.7 kg/m² in studies that reported. |
|  | C6. What types of bariatric surgery were included? | Gastric bypass (most common), Sleeve Gastrectomy (SG), Vertical Banded Gastroplasty (VBG), Gastric plication, and Adjustable Gastric Band (AGB). |
| D. UDCA DOSING PROTOCOL | D1. What UDCA dosage was used? | Variable among studies. Commonly 500-600 mg/day. Some studies used 1000 mg/day or 1200 mg/day. |
|  | D2. Was the dosage based on body weight? | Not explicitly reported for the meta-analysis, but one study used 10 mg/kg/day. |
|  | D3. If so, what was the dosage per kg? | Variable (10 mg/kg/day in one study, otherwise not applicable). |
|  | D4. What was the daily administration frequency? | Once or twice daily. |
|  | D5. Was the dosage adjusted during treatment? | Not reported in the meta-analysis, but mentioned that higher doses (1200mg) were associated with lower adherence. |
|  | D6. If so, what were the criteria for adjustment? | Not applicable. |
| E. TREATMENT DURATION PROTOCOL | E1. What was the total duration of UDCA treatment? | Variable among studies, generally 6 months. |
|  | E2. Was the duration predefined or criteria-based? | Predefined (in most original studies). |
|  | E3. If criteria-based, what were they? | Not applicable. |
|  | E4. Did any patients discontinue earlier than planned? | Yes (variably among studies). |
|  | E5. If so, what were the reasons for discontinuation? | Adverse events and non-adherence. Low adherence (28%) reported in one study. |
|  | E6. What was the protocol adherence rate? | Variable among studies. One study reported 89% adherence in the UDCA group. Higher doses were associated with lower adherence. |
| F. INITIATION TIMING PROTOCOL | F1. When was UDCA initiated in relation to surgery? | Postoperatively. |
|  | F2. If preoperatively, how many days before surgery? | Not applicable. |
|  | F3. If postoperatively, how many days after surgery? | Variable. Most studies initiated UDCA "within 10 days postoperatively," one study "6 weeks postoperatively," and another "when the patient recovered." |
|  | F4. Was the initiation timing standardized for all patients? | Not standardized among the studies in the meta-analysis. |
|  | F5. Were there specific criteria for determining initiation? | Not applicable. |
|  | F6. If so, what criteria were used? | Not applicable. |
| G. MONITORING AND FOLLOW-UP | G1. Was monitoring performed during UDCA use? | Yes (in the original studies). |
|  | G2. If so, what was the monitoring frequency? | Variable. Assessments at 3, 6, and 12 months were common. |
|  | G3. What tests were used for monitoring? | Not detailed in the meta-analysis, but in primary studies: ultrasound examinations. |
|  | G4. How was gallstone formation assessed? | Ultrasonography. |
|  | G5. What was the frequency of cholelithiasis assessment? | Variable among studies (3, 6, 12, 18, 24 months). |
|  | G6. Were there predefined criteria to discontinue UDCA? | Not reported in the meta-analysis, but in primary studies: development of symptoms, adverse events. |
| H. EFFICACY OUTCOMES | H1. What was the incidence of cholelithiasis in the UDCA group? | 7.3% (overall). |
|  | H2. What was the incidence of cholelithiasis in the control group (if applicable)? | 24.7% (overall). |
|  | H3. What was the mean time to cholelithiasis development? | UDCA significantly reduced gallstone formation at all time intervals (3, 6, 12 months). 5.3% at 3 months, 6.8% at 6 months, 8.6% at 12 months. |
|  | H4. Was there a statistically significant difference? | Yes (RR 0.36; P < 0.00001). |
|  | H5. What was the calculated preventive efficacy? | 64% risk reduction. |
|  | H6. How many patients required cholecystectomy? | The exact number not reported, but symptomatic gallstone formation was significantly lower (RR 0.24; P < 0.00001), implying less need for cholecystectomy. |
| I. SAFETY AND ADVERSE EVENTS | I1. Were UDCA-related adverse events reported? | Yes (in the original studies). |
|  | I2. If so, what were the main adverse events? | Diarrhea, nausea/vomiting, abdominal pain, headaches/dizziness, rashes. |
|  | I3. What was the incidence of adverse events? | Varied from 4.8% to 20.9% in the studies. |
|  | I4. Was there discontinuation due to adverse events? | Yes (in some studies). |
|  | I5. If so, what was the discontinuation rate? | No consolidated rate reported, but one study reported 9/86 (10%) due to medication intolerance. |
|  | K1. What limitations were reported by the authors? | Significant heterogeneity among studies; high dropout rate and/or low adherence in primary studies. |
|  | K2. Were conflicts of interest declared? | No ("The authors have no commercial associations that could be a conflict of interest"). |
|  | K3. What was the loss to follow-up rate? | Variable among primary studies. One study reported a "high dropout rate" at 12 months. |
|  | K4. Did the study have adequate statistical power? | Not specified for the meta-analysis, but primary studies are RCTs. |
|  | K5. Were appropriate randomization methods used (if RCT)? | Yes (Meta-analysis of RCTs). |
|  | K6. Was adequate blinding used (if applicable)? | Yes (Meta-analysis of RCTs, most were double-blind). |

Table S5. Data extraction details - Vural et al., 2020

| Domain | Extraction Questions | Detailed Answers |
| --- | --- | --- |
| A. STUDY IDENTIFICATION | A1. First author and year of publication | Vural et al., 2020 |
|  | A2. Study title | Increased gallstone formation after sleeve gastrectomy and the preventive role of ursodeoxycholic acid |
|  | A3. Publishing journal | Acta gastroenterol. belg. |
|  | A4. Country/region where conducted | Turkey |
|  | A5. Language of publication | English |
|  | A6. Funding source | Not reported (The authors declare no conflicts of interest.) |
| B. METHODOLOGICAL CHARACTERISTICS | B1. What was the study design? | Retrospective cohort study. |
|  | B2. What was the data collection period? | March 2016 to March 2018. |
|  | B3. What was the follow-up duration? | Between 6 and 18 months (control ultrasound between 6 and 18 months). |
|  | B4. Was the study multicentric? | No (Single hospital: Fatih Sultan Mehmet Training and Research Hospital) |
|  | B5. What was the study setting? | Hospital |
| C. STUDY POPULATION | C1. What was the total sample size? | 108 patients included in the analysis. |
|  | C2. How many patients received UDCA? | 42 patients. |
|  | C3. What was the mean/median age of patients? | 39.62 ± 10.62 years (range 19 to 66 years). |
|  | C4. What was the percentage of female patients? | Not specifically reported, but tables indicate 18 (38%) women in the gallstone group and 25 (53%) in the no gallstone group in the UDCA subgroup. And 25 (41%) without UDCA and no gallstones. Totals for each group need to be summed. (Total 108 patients, distributed into "Developed Gallstones" (n=47) and "No Gallstones" (n=61)). For the UDCA group (n=42): 24 women (57.1%). For the No UDCA group (n=66): 48 women (72.7%). Total women: 72 (66.7%). |
|  | C5. What was the mean/median preoperative BMI? | Not reported directly for the UDCA group, but mean initial BMI in Table 1 is 45.87 ± 5.4 kg/m² for gallstone group (UDCA and non-UDCA) and 44.18 ± 4.2 kg/m² for no gallstone group (UDCA and non-UDCA). |
|  | C6. What types of bariatric surgery were included? | Sleeve Gastrectomy (SG). |
| D. UDCA DOSING PROTOCOL | D1. What UDCA dosage was used? | 500 mg/day. |
|  | D2. Was the dosage based on body weight? | No. |
|  | D3. If so, what was the dosage per kg? | Not applicable. |
|  | D4. What was the daily administration frequency? | Twice daily (2x250 mg). |
|  | D5. Was the dosage adjusted during treatment? | Not reported. |
|  | D6. If so, what were the criteria for adjustment? | Not applicable. |
| E. TREATMENT DURATION PROTOCOL | E1. What was the total duration of UDCA treatment? | Not explicitly reported in the methods section, but the introduction states that "decided to systematically prescribe UDCA in the first 6 postoperative months" and the Discussion section mentions "UDCA 500 mg/d for 6 months". Follow-up is 6-18 months. |
|  | E2. Was the duration predefined or criteria-based? | Predefined (6 months is a common period, but not explicitly stated for this study as treatment duration in Methods). |
|  | E3. If criteria-based, what were they? | Not applicable. |
|  | E4. Did any patients discontinue earlier than planned? | Not reported. |
|  | E5. If so, what were the reasons for discontinuation? | Not applicable. |
|  | E6. What was the protocol adherence rate? | Not reported. |
| F. INITIATION TIMING PROTOCOL | F1. When was UDCA initiated in relation to surgery? | Postoperatively. |
|  | F2. If preoperatively, how many days before surgery? | Not applicable. |
|  | F3. If postoperatively, how many days after surgery? | Not specified in exact days, but "Patients who underwent bariatric surgery after July 2017 were given prophylactically a daily dose of 2x250 mg UDCA". |
|  | F4. Was the initiation timing standardized for all patients? | Yes (for patients operated after July 2017). |
|  | F5. Were there specific criteria for determining initiation? | No (decision to change clinical practice). |
|  | F6. If so, what criteria were used? | Not applicable. |
| G. MONITORING AND FOLLOW-UP | G1. Was monitoring performed during UDCA use? | Yes. |
|  | G2. If so, what was the monitoring frequency? | Not specified. Only "returned to our clinic for a control ultrasound between 6 and 18 months". |
|  | G3. What tests were used for monitoring? | Serum lipid profile (Triglycerides, Total Cholesterol, HDL, LDL) and BMI. (In addition to ultrasound). |
|  | G4. How was gallstone formation assessed? | Abdominal ultrasonography (performed by a researcher). |
|  | G5. What was the frequency of cholelithiasis assessment? | Between 6 and 18 months after the operation. |
|  | G6. Were there predefined criteria to discontinue UDCA? | Not reported. |
| H. EFFICACY OUTCOMES | H1. What was the incidence of cholelithiasis in the UDCA group? | 10.18% (11 of 42 patients who took UDCA developed gallstones). |
|  | H2. What was the incidence of cholelithiasis in the control group (if applicable)? | 33.33% (36 of 66 patients who did not take UDCA developed gallstones). |
|  | H3. What was the mean time to cholelithiasis development? | Not specifically reported. |
|  | H4. Was there a statistically significant difference? | Yes (P = 0.007). |
|  | H5. What was the calculated preventive efficacy? | Significant reduction: (from 33.33% to 10.18%). |
|  | H6. How many patients required cholecystectomy? | Not reported. |
| I. SAFETY AND ADVERSE EVENTS | I1. Were UDCA-related adverse events reported? | Not reported. |
|  | I2. If so, what were the main adverse events? | Not applicable. |
|  | I3. What was the incidence of adverse events? | Not applicable. |
|  | I4. Was there discontinuation due to adverse events? | Not reported. |
|  | I5. If so, what was the discontinuation rate? | Not applicable. |
|  | I6. Were contraindications to use reported? | Not reported. |
| J. VARIATIONS BY SURGERY TYPE | J1. Did the protocol vary according to the type of bariatric surgery? | No, only SG was included. |
|  | J2. If so, what were the differences by surgery type? | Not applicable. |
|  | J3. Was there justification for protocol variations? | Not applicable. |
|  | J4. Which surgery type showed the greatest benefit? | Not applicable. |
|  | J5. Were specific recommendations reported per procedure? | Not applicable. |
| K. LIMITATIONS AND QUALITY | K1. What limitations were reported by the authors? | Not explicitly reported in the discussion. (The study is retrospective, which is an inherent limitation). |
|  | K2. Were conflicts of interest declared? | No ("Authors declare no conflict of interest"). |
|  | K3. What was the loss to follow-up rate? | 24 patients (14.2%) were excluded for not attending postoperative controls (US and lab tests). |
|  | K4. Did the study have adequate statistical power? | Not specified. |
|  | K5. Were appropriate randomization methods used (if RCT)? | Not applicable (not randomized). |
|  | K6. Was adequate blinding used (if applicable)? | Not applicable (not blinded). |

Table S6. Data extraction details - Machado et al., 2019

| Domain | Extraction Questions | Detailed Answers |
| --- | --- | --- |
| A. STUDY IDENTIFICATION | A1. First author and year of publication | Machado et al., 2019 |
|  | A2. Study title | Ursodeoxycholic acid in the prevention of gallstones in patients subjected to Roux-en-Y gastric bypass |
|  | A3. Publishing journal | Acta Cirúrgica Brasileira |
|  | A4. Country/region where conducted | Brazil |
|  | A5. Language of publication | English |
|  | A6. Funding source | Brazilian Ministry of Health, and CNPq |
| B. METHODOLOGICAL CHARACTERISTICS | B1. What was the study design? | Comparative, prospective, community-based clinical study (comparison of two non-randomized groups) |
|  | B2. What was the data collection period? | April 2015 to August 2016 (patients operated between January 2014 and December 2015). |
|  | B3. What was the follow-up duration? | Minimum of 6 months after surgery (follow-up period for all patients). |
|  | B4. Was the study multicentric? | No (two clinics providing bariatric surgery service) |
|  | B5. What was the study setting? | Outpatient/hospital clinics |
| C. STUDY POPULATION | C1. What was the total sample size? | 137 patients. |
|  | C2. How many patients received UDCA? | 69 patients (Group A). |
|  | C3. What was the mean/median age of patients? | Group A: 34.18 (SD = 13.36) years; Group B (Control): 35.91 (SD = 10.28) years. |
|  | C4. What was the percentage of female patients? | Group A: 73.9% (51/69); Group B (Control): 60.3% (41/68). |
|  | C5. What was the mean/median preoperative BMI? | Group A: 41.80 (SD = 5.00) kg/m²; Group B (Control): 42.20 (SD = 5.80) kg/m². |
|  | C6. What types of bariatric surgery were included? | Roux-en-Y Gastric Bypass (videolaparoscopy). |
| D. UDCA DOSING PROTOCOL | D1. What UDCA dosage was used? | 300 mg/day. |
|  | D2. Was the dosage based on body weight? | No. |
|  | D3. If so, what was the dosage per kg? | Not applicable. |
|  | D4. What was the daily administration frequency? | Twice daily (150 mg twice daily). |
|  | D5. Was the dosage adjusted during treatment? | Not reported. |
|  | D6. If so, what were the criteria for adjustment? | Not applicable. |
| E. TREATMENT DURATION PROTOCOL | E1. What was the total duration of UDCA treatment? | 5 consecutive months. |
|  | E2. Was the duration predefined or criteria-based? | Predefined. |
|  | E3. If criteria-based, what were they? | Not applicable. |
|  | E4. Did any patients discontinue earlier than planned? | No (100% of patients adhered to medication use in Group A). |
|  | E5. If so, what were the reasons for discontinuation? | Not applicable. |
|  | E6. What was the protocol adherence rate? | 100% (for the group that received UDCA). |
| F. INITIATION TIMING PROTOCOL | F1. When was UDCA initiated in relation to surgery? | Postoperatively. |
|  | F2. If preoperatively, how many days before surgery? | Not applicable. |
|  | F3. If postoperatively, how many days after surgery? | 30 days after surgery. |
|  | F4. Was the initiation timing standardized for all patients? | Yes (30 days after surgery for all in Group A). |
|  | F5. Were there specific criteria for determining initiation? | No (protocol standardization). |
|  | F6. If so, what criteria were used? | Not applicable. |
| G. MONITORING AND FOLLOW-UP | G1. Was monitoring performed during UDCA use? | Yes. |
|  | G2. If so, what was the monitoring frequency? | Not specified for monitoring frequency during treatment, but ultrasound to assess cholelithiasis was performed. |
|  | G3. What tests were used for monitoring? | Not specified for UDCA monitoring. (For initial eligibility: full assessment by multidisciplinary team). |
|  | G4. How was gallstone formation assessed? | Abdominal ultrasonography. |
|  | G5. What was the frequency of cholelithiasis assessment? | Once, 6 months after surgery. |
|  | G6. Were there predefined criteria to discontinue UDCA? | Not reported. |
| H. EFFICACY OUTCOMES | H1. What was the incidence of cholelithiasis in the UDCA group? | 1.4% (1 of 69 patients). |
|  | H2. What was the incidence of cholelithiasis in the control group (if applicable)? | 26.4% (18 of 68 patients). |
|  | H3. What was the mean time to cholelithiasis development? | Not reported an exact mean time. |
|  | H4. Was there a statistically significant difference? | Yes (OR = 24.4, P < 0.001). |
|  | H5. What was the calculated preventive efficacy? | Significant reduction (from 26.4% to 1.4%). |
|  | H6. How many patients required cholecystectomy? | Not reported. |
| I. SAFETY AND ADVERSE EVENTS | I1. Were UDCA-related adverse events reported? | Not explicitly reported in the results or discussion section. |
|  | I2. If so, what were the main adverse events? | Not applicable. |
|  | I3. What was the incidence of adverse events? | Not applicable. |
|  | I4. Was there discontinuation due to adverse events? | Not reported. (100% adherence suggests few impactful adverse events). |
|  | I5. If so, what was the discontinuation rate? | Not applicable. |
|  | I6. Were contraindications to use reported? | Not explicitly reported. Exclusion criteria included "pregnant women", "participants in other investigations with investigative drugs", "those unable to sign informed consent form". |
| J. VARIATIONS BY SURGERY TYPE | J1. Did the protocol vary according to the type of bariatric surgery? | No, only Roux-en-Y Gastric Bypass. |
|  | J2. If so, what were the differences by surgery type? | Not applicable. |
|  | J3. Was there justification for protocol variations? | Not applicable. |
|  | J4. Which surgery type showed the greatest benefit? | Not applicable. |
|  | J5. Were specific recommendations reported per procedure? | Not applicable. |
| K. LIMITATIONS AND QUALITY | K1. What limitations were reported by the authors? | The study was conducted through a community clinical trial, which could have introduced selection bias and confounding. No placebo was used in the control group. |
|  | K2. Were conflicts of interest declared? | No ("Conflict of interest: none"). |
|  | K3. What was the loss to follow-up rate? | Not reported directly, but patients were "allocated into two groups: 68 patients in GROUP B and 69 patients in GROUP A". |
|  | K4. Did the study have adequate statistical power? | Not specified. |
|  | K5. Were appropriate randomization methods used (if RCT)? | No (groups were allocated "according to the clinic of reference", not by randomization). |
|  | K6. Was adequate blinding used (if applicable)? | Not reported (inferred that there was no blinding). |

Table S7. Data extraction details - Al-huniti et al., 2023

| Domain | Extraction Questions | Detailed Answers |
| --- | --- | --- |
| A. STUDY IDENTIFICATION | A1. First author and year of publication | Al-huniti et al., 2023 |
|  | A2. Study title | Ursodeoxycholic Acid Prophylaxis and the Reduction of Gallstone Formation After Bariatric Surgery: An Updated Meta-Analysis of Randomized Controlled Trials |
|  | A3. Publishing journal | Cureus |
|  | A4. Country/region where conducted | Not applicable (Global Meta-analysis) |
|  | A5. Language of publication | English |
|  | A6. Funding source | Not reported (The authors stated that they did not receive financial support from any organization for the submitted work.) |
| B. METHODOLOGICAL CHARACTERISTICS | B1. What was the study design? | Systematic review and Meta-analysis of Randomized Clinical Trials (RCTs) |
|  | B2. What was the data collection period? | Literature search until September 2023. Included articles published between 1993 and 2022. |
|  | B3. What was the follow-up duration? | Variable among included studies (maximum 1 year for gallstone incidence, but follow-up may be longer for other outcomes). |
|  | B4. Was the study multicentric? | Not applicable (Meta-analysis of studies) |
|  | B5. What was the study setting? | Not applicable (Meta-analysis of studies) |
| C. STUDY POPULATION | C1. What was the total sample size? | 2767 patients in the 12 included RCTs. |
|  | C2. How many patients received UDCA? | 1881 patients (approximately, summing "Sample size per group AUDC" from Table 1). |
|  | C3. What was the mean/median age of patients? | No significant difference in age between groups was found (SMD = 0.23; P = 0.82). |
|  | C4. What was the percentage of female patients? | No significant difference in gender between groups was found (RR = 0.51; P = 0.61). |
|  | C5. What was the mean/median preoperative BMI? | No significant difference in baseline BMI between groups was found (SMD = 0.91; P = 0.36). |
|  | C6. What types of bariatric surgery were included? | Not explicitly specified for all RCTs, but the review's guiding question includes "different types of bariatric surgery." Meta-analyses generally cover RYGB and SG. |
| D. UDCA DOSING PROTOCOL | D1. What UDCA dosage was used? | Variable among primary studies (not consolidated in the meta-analysis). |
|  | D2. Was the dosage based on body weight? | Not reported for the meta-analysis. |
|  | D3. If so, what was the dosage per kg? | Not reported for the meta-analysis. |
|  | D4. What was the daily administration frequency? | Variable among primary studies. |
|  | D5. Was the dosage adjusted during treatment? | Not reported for the meta-analysis. |
|  | D6. If so, what were the criteria for adjustment? | Not applicable. |
| E. TREATMENT DURATION PROTOCOL | E1. What was the total duration of UDCA treatment? | Variable among primary studies. |
|  | E2. Was the duration predefined or criteria-based? | Predefined in primary studies. |
|  | E3. If criteria-based, what were they? | Not applicable. |
|  | E4. Did any patients discontinue earlier than planned? | Not reported in the meta-analysis. |
|  | E5. If so, what were the reasons for discontinuation? | Not applicable. |
|  | E6. What was the protocol adherence rate? | Not reported in the meta-analysis. |
| F. INITIATION TIMING PROTOCOL | F1. When was UDCA initiated in relation to surgery? | Postoperatively (inferred, as it is postoperative prophylaxis). |
|  | F2. If preoperatively, how many days before surgery? | Not applicable. |
|  | F3. If postoperatively, how many days after surgery? | Not reported for the meta-analysis. |
|  | F4. Was the initiation timing standardized for all patients? | Not standardized among primary studies. |
|  | F5. Were there specific criteria for determining initiation? | Not applicable. |
|  | F6. If so, what criteria were used? | Not applicable. |
| G. MONITORING AND FOLLOW-UP | G1. Was monitoring performed during UDCA use? | Yes (in primary studies). |
|  | G2. If so, what was the monitoring frequency? | Variable. |
|  | G3. What tests were used for monitoring? | Ultrasonography (mainly). |
|  | G4. How was gallstone formation assessed? | Ultrasonography. |
|  | G5. What was the frequency of cholelithiasis assessment? | At 3, 6, and 12 months (for meta-analysis sub-analysis). |
|  | G6. Were there predefined criteria to discontinue UDCA? | Not reported in the meta-analysis. |
| H. EFFICACY OUTCOMES | H1. What was the incidence of cholelithiasis in the UDCA group? | The meta-analysis showed that patients receiving UDCA have a lower overall incidence of gallstones (RR = 0.13; P < 0.0001). |
|  | H2. What was the incidence of cholelithiasis in the control group (if applicable)? | The control group had a higher incidence. |
|  | H3. What was the mean time to cholelithiasis development? | Incidence of gallstones assessed at 3, 6, and 12 months. UDCA reduced incidence at all these periods (P = 0.04, P < 0.00001 and P < 0.00001, respectively). |
|  | H4. Was there a statistically significant difference? | Yes (P < 0.0001 for overall incidence). |
|  | H5. What was the calculated preventive efficacy? | 87% reduction in the relative risk of gallstone formation. |
|  | H6. How many patients required cholecystectomy? | Lower cholecystectomy rate in the UDCA group (RR = 3.05; P = 0.002). |
| I. SAFETY AND ADVERSE EVENTS | I1. Were UDCA-related adverse events reported? | Not detailed in the meta-analysis, but the general discussion of primary studies may have addressed this. |
|  | I2. If so, what were the main adverse events? | Not reported in the meta-analysis. |
|  | I3. What was the incidence of adverse events? | Not reported in the meta-analysis. |
|  | I4. Was there discontinuation due to adverse events? | Not reported in the meta-analysis. |
|  | I5. If so, what was the discontinuation rate? | Not applicable. |
|  | I6. Were contraindications to use reported? | Not reported in the meta-analysis. |
| J. VARIATIONS BY SURGERY TYPE | J1. Did the protocol vary according to the type of bariatric surgery? | Yes, primary studies included various types (gastric bypass, sleeve gastrectomy). |
|  | J2. If so, what were the differences by surgery type? | Not detailed in the meta-analysis. |
|  | J3. Was there justification for protocol variations? | Not applicable. |
|  | J4. Which surgery type showed the greatest benefit? | Not detailed in the meta-analysis. |
|  | J5. Were specific recommendations reported per procedure? | Not applicable. |
| K. LIMITATIONS AND QUALITY | K1. What limitations were reported by the authors? | Heterogeneity in primary studies (design, surgical procedures, UDCA regimens); limited follow-up duration (up to 1 year for some outcomes); patient heterogeneity; potential for selection and publication bias. |
|  | K2. Were conflicts of interest declared? | No ("The authors declared that there are no conflicts of interest"). |
|  | K3. What was the loss to follow-up rate? | Not consolidated in the meta-analysis. |
|  | K4. Did the study have adequate statistical power? | Not applicable (meta-analysis). |
|  | K5. Were appropriate randomization methods used (if RCT)? | Yes (meta-analysis of RCTs). |
|  | K6. Was adequate blinding used (if applicable)? | Yes (meta-analysis of RCTs, generally blinded). |

Table S8. Data extraction details - Stokes et al., 2014

| Domain | Extraction Questions | Detailed Answers |
| --- | --- | --- |
| A. STUDY IDENTIFICATION | A1. First author and year of publication | Stokes et al., 2014 |
|  | A2. Study title | Ursodeoxycholic Acid and Diets Higher in Fat Prevent Gallbladder Stones During Weight Loss: A Meta-analysis of Randomized Controlled Trials |
|  | A3. Publishing journal | Clinical Gastroenterology and Hepatology |
|  | A4. Country/region where conducted | Not applicable (Global Meta-analysis) |
|  | A5. Language of publication | English |
|  | A6. Funding source | Not reported (Conflict of interest statement: Frank Lammert received lecture fees from Falk Foundation e.V. Lise Gluud participated in a Merck-funded trial. The other authors declare no conflicts.) |
| B. METHODOLOGICAL CHARACTERISTICS | B1. What was the study design? | Systematic review and Meta-analysis of Randomized Clinical Trials (RCTs) |
|  | B2. What was the data collection period? | Literature search until July 2013. Included articles published from 1988 to 2003. |
|  | B3. What was the follow-up duration? | Variable among included studies (minimum 6 weeks to 24 months). |
|  | B4. Was the study multicentric? | Not applicable (Meta-analysis of studies) |
|  | B5. What was the study setting? | Not applicable (Meta-analysis of studies) |
| C. STUDY POPULATION | C1. What was the total sample size? | 1836 participants in the 13 RCTs (8 diet, 5 bariatric surgery). |
|  | C2. How many patients received UDCA? | Not specified separately for UDCA (but 11 studies evaluated UDCA). |
|  | C3. What was the mean/median age of patients? | Not consolidated, but participants were obese (BMI > 30 kg/m²). |
|  | C4. What was the percentage of female patients? | Variable (range 42% to 100% female). |
|  | C5. What was the mean/median preoperative BMI? | Not consolidated, but bariatric surgery studies had a median baseline BMI of 143 kg (vs 103 kg for diets). |
|  | C6. What types of bariatric surgery were included? | Bariatric surgery (Gastric Bypass, Vertical Banded Gastroplasty, Adjustable Gastric Band) and diet interventions for weight loss. |
| D. UDCA DOSING PROTOCOL | D1. What UDCA dosage was used? | Variable among studies. 300–1200 mg/day (median of 750 mg/day). |
|  | D2. Was the dosage based on body weight? | One study used 10 mg/kg/day (Williams, 1993). |
|  | D3. If so, what was the dosage per kg? | 10 mg/kg/day in one study. |
|  | D4. What was the daily administration frequency? | Variable (not consolidated in the meta-analysis). |
|  | D5. Was the dosage adjusted during treatment? | Not reported in the meta-analysis. |
|  | D6. If so, what were the criteria for adjustment? | Not applicable. |
| E. TREATMENT DURATION PROTOCOL | E1. What was the total duration of UDCA treatment? | Variable among studies, from 6 weeks to 18 months. |
|  | E2. Was the duration predefined or criteria-based? | Predefined in primary studies. |
|  | E3. If criteria-based, what were they? | Not applicable. |
|  | E4. Did any patients discontinue earlier than planned? | Yes (in some studies). |
|  | E5. If so, what were the reasons for discontinuation? | Not reported in the meta-analysis. |
|  | E6. What was the protocol adherence rate? | Not reported in the meta-analysis, but discussion suggests poor compliance can be an issue. |
| F. INITIATION TIMING PROTOCOL | F1. When was UDCA initiated in relation to surgery? | Postoperative for bariatric surgery studies; immediately at the start of caloric restriction for diet studies. |
|  | F2. If preoperatively, how many days before surgery? | Not applicable (for bariatric surgery). |
|  | F3. If postoperatively, how many days after surgery? | Variable in bariatric surgery studies (within days, or 6 weeks). |
|  | F4. Was the initiation timing standardized for all patients? | Not standardized among primary studies. |
|  | F5. Were there specific criteria for determining initiation? | Not applicable. |
|  | F6. If so, what criteria were used? | Not applicable. |
| G. MONITORING AND FOLLOW-UP | G1. Was monitoring performed during UDCA use? | Yes (in primary studies). |
|  | G2. If so, what was the monitoring frequency? | Variable (minimum of 6 weeks to 24 months). |
|  | G3. What tests were used for monitoring? | Ultrasonography (mainly). |
|  | G4. How was gallstone formation assessed? | Ultrasonography. |
|  | G5. What was the frequency of cholelithiasis assessment? | Variable among studies. |
|  | G6. Were there predefined criteria to discontinue UDCA? | Not reported in the meta-analysis. |
| H. EFFICACY OUTCOMES | H1. What was the incidence of cholelithiasis in the UDCA group? | 5% (62 of 1217 participants). |
|  | H2. What was the incidence of cholelithiasis in the control group (if applicable)? | 23% (130 of 574 participants). |
|  | H3. What was the mean time to cholelithiasis development? | Not consolidated, but the critical period is within the first 6 months after surgery. |
|  | H4. Was there a statistically significant difference? | Yes (RR = 0.33; 95% CI, 0.18–0.60). |
|  | H5. What was the calculated preventive efficacy? | 67% reduction in the relative risk of gallstone formation. Number Needed to Treat (NNT) = 9. |
|  | H6. How many patients required cholecystectomy? | UDCA reduced the risk of cholecystectomy for symptomatic gallstones (RR = 0.20; 95% CI, 0.07–0.53). NNT = 15. |
| I. SAFETY AND ADVERSE EVENTS | I1. Were UDCA-related adverse events reported? | Yes (in primary studies). |
|  | I2. If so, what were the main adverse events? | Gastrointestinal complaints (abdominal pain, nausea, diarrhea, constipation), headache, dizziness, rashes. |
|  | I3. What was the incidence of adverse events? | Not consolidated, but few serious events. One study (Scott, 2003) reported 25% AEs in the UDCA group. |
|  | I4. Was there discontinuation due to adverse events? | Yes (in some studies). |
|  | I5. If so, what was the discontinuation rate? | Not consolidated, but 13 patients withdrawn from one study (Shiffman, 1995) due to AEs. |
|  | I6. Were contraindications to use reported? | Not reported in the meta-analysis. |
| J. VARIATIONS BY SURGERY TYPE | J1. Did the protocol vary according to the type of bariatric surgery? | Yes, there were diet and bariatric surgery studies. |
|  | J2. If so, what were the differences by surgery type? | Diet studies: RR 0.17 (greater benefit). Bariatric surgery studies: RR 0.42 (lesser benefit). |
|  | J3. Was there justification for protocol variations? | Not applicable (variations reflect the diversity of primary studies). |
|  | J4. Which surgery type showed the greatest benefit? | The benefit of UDCA was "significantly greater" in isolated diet studies compared to bariatric surgery studies (test for subgroup differences, P = 0.03). |
|  | J5. Were specific recommendations reported per procedure? | Not applicable. |
| K. LIMITATIONS AND QUALITY | K1. What limitations were reported by the authors? | Small number of studies and small samples for some meta-analyses; high attrition bias rate in some primary studies; quality of life measures were not addressed. |
|  | K2. Were conflicts of interest declared? | Yes (F. Lammert, L. Gluud). |
|  | K3. What was the loss to follow-up rate? | High in some primary studies. |
|  | K4. Did the study have adequate statistical power? | Not applicable (meta-analysis). The sequential trial analysis did not confirm the robustness of the results due to sparse data and multiple testing. |
|  | K5. Were appropriate randomization methods used (if RCT)? | Yes (meta-analysis of RCTs). |
|  | K6. Was adequate blinding used (if applicable)? | Yes (most primary studies were double-blind). |

Table S9. Data extraction details - Salman et al., 2022

| Domain | Extraction Questions | Detailed Answers |
| --- | --- | --- |
| A. STUDY IDENTIFICATION | A1. First author and year of publication | Salman et al., 2022 |
|  | A2. Study title | Ursodeoxycholic acid for the prevention of gall stones after laparoscopic sleeve gastrectomy: a prospective controlled study |
|  | A3. Publishing journal | Surgical Endoscopy |
|  | A4. Country/region where conducted | Not explicitly reported (inferred Egypt, given that the authors of the Nabil et al. 2019 article are from Egypt and this is a complementary article or from the same group) |
|  | A5. Language of publication | English |
|  | A6. Funding source | Not reported (The authors have no conflicts of interest or financial ties to disclose.) |
| B. METHODOLOGICAL CHARACTERISTICS | B1. What was the study design? | Randomized Clinical Trial (RCT) and Prospective. |
|  | B2. What was the data collection period? | June 2017 to June 2019. |
|  | B3. What was the follow-up duration? | 12 months. |
|  | B4. Was the study multicentric? | Not explicitly reported, but several authors from different departments and locations, suggesting possible multicentricity or collaboration. |
|  | B5. What was the study setting? | Hospital (patients scheduled for LSG). |
| C. STUDY POPULATION | C1. What was the total sample size? | 332 patients initially scheduled, 258 patients included in the final analysis. |
|  | C2. How many patients received UDCA? | 130 patients (UDCA group). |
|  | C3. What was the mean/median age of patients? | UDCA group: 39.3 ± 9.0 years; Control group: 34.7 ± 9.9 years. |
|  | C4. What was the percentage of female patients? | UDCA group: 66.9% (87/130); Control group: 60.9% (78/128). |
|  | C5. What was the mean/median preoperative BMI? | UDCA group: 42.9 ± 3.6 kg/m²; Control group: 43.3 ± 3.6 kg/m². |
|  | C6. What types of bariatric surgery were included? | Laparoscopic Sleeve Gastrectomy (LSG). |
| D. UDCA DOSING PROTOCOL | D1. What UDCA dosage was used? | 500 mg/day. |
|  | D2. Was the dosage based on body weight? | No. |
|  | D3. If so, what was the dosage per kg? | Not applicable. |
|  | D4. What was the daily administration frequency? | Once daily. |
|  | D5. Was the dosage adjusted during treatment? | Not reported. |
|  | D6. If so, what were the criteria for adjustment? | Not applicable. |
| E. TREATMENT DURATION PROTOCOL | E1. What was the total duration of UDCA treatment? | 12 months. |
|  | E2. Was the duration predefined or criteria-based? | Predefined. |
|  | E3. If criteria-based, what were they? | Not applicable. |
|  | E4. Did any patients discontinue earlier than planned? | Yes (3 patients in the UDCA group). |
|  | E5. If so, what were the reasons for discontinuation? | Severe adverse events (allergy and severe gastrointestinal symptoms). |
|  | E6. What was the protocol adherence rate? | Not explicitly reported, but 3 out of 133 patients discontinued due to AEs, which would be approximately 2.2%. |
| F. INITIATION TIMING PROTOCOL | F1. When was UDCA initiated in relation to surgery? | Postoperatively. |
|  | F2. If preoperatively, how many days before surgery? | Not applicable. |
|  | F3. If postoperatively, how many days after surgery? | Not specified in exact days, but "for 12 months postoperatively". |
|  | F4. Was the initiation timing standardized for all patients? | Yes (postoperative prophylaxis regimen). |
|  | F5. Were there specific criteria for determining initiation? | No (systematic prescription). |
|  | F6. If so, what criteria were used? | Not applicable. |
| G. MONITORING AND FOLLOW-UP | G1. Was monitoring performed during UDCA use? | Yes. |
|  | G2. If so, what was the monitoring frequency? | Not specified for UDCA use monitoring. (Gallstone monitoring: at 6 and 12 months). |
|  | G3. What tests were used for monitoring? | Ultrasonography (to detect gallstones). |
|  | G4. How was gallstone formation assessed? | Ultrasonography. |
|  | G5. What was the frequency of cholelithiasis assessment? | At 6 and 12 months after surgery. |
|  | G6. Were there predefined criteria to discontinue UDCA? | Yes (in case of severe adverse events). |
| H. EFFICACY OUTCOMES | H1. What was the incidence of cholelithiasis in the UDCA group? | 8.5% (11 of 130 patients) in the first postoperative year. |
|  | H2. What was the incidence of cholelithiasis in the control group (if applicable)? | 32.0% (41 of 128 patients) in the first postoperative year. |
|  | H3. What was the mean time to cholelithiasis development? | Not reported an exact mean time. Assessed at 6 and 12 months. |
|  | H4. Was there a statistically significant difference? | Yes (P < 0.001). |
|  | H5. What was the calculated preventive efficacy? | Significant reduction (from 32% to 8.5%). |
|  | H6. How many patients required cholecystectomy? | 3 patients (2.3%) in the UDCA group and 9 patients (7.0%) in the Control group (P = 0.072). |
| I. SAFETY AND ADVERSE EVENTS | I1. Were UDCA-related adverse events reported? | Yes. |
|  | I2. If so, what were the main adverse events? | Allergy and severe gastrointestinal symptoms. |
|  | I3. What was the incidence of adverse events? | 3 patients (approximately 2.2% of the original UDCA group of 133 patients). |
|  | I4. Was there discontinuation due to adverse events? | Yes (3 patients). |
|  | I5. If so, what was the discontinuation rate? | 2.2%. |
|  | I6. Were contraindications to use reported? | Not explicitly reported. Exclusion criteria included "preoperative gallstones and abnormal liver function tests or previous history of cholecystectomy". |
| J. VARIATIONS BY SURGERY TYPE | J1. Did the protocol vary according to the type of bariatric surgery? | No, only LSG was included. |
|  | J2. If so, what were the differences by surgery type? | Not applicable. |
|  | J3. Was there justification for protocol variations? | Not applicable. |
|  | J4. Which surgery type showed the greatest benefit? | Not applicable. |
|  | J5. Were specific recommendations reported per procedure? | Not applicable. |
| K. LIMITATIONS AND QUALITY | K1. What limitations were reported by the authors? | Lack of data on gallstone size; absence of standardization of postoperative dietary habits (potential contributing factor); lack of details on the study population in the geographical area. |
|  | K2. Were conflicts of interest declared? | No ("The authors have no conflicts of interest or financial ties to disclose"). |
|  | K3. What was the loss to follow-up rate? | 71 patients (21.4%) were lost to follow-up (33 from the UDCA group and 38 from the Control group). |
|  | K4. Did the study have adequate statistical power? | Not specified in the text, but it is a prospective RCT. |
|  | K5. Were appropriate randomization methods used (if RCT)? | Yes (simple randomization using sealed envelopes). |
|  | K6. Was adequate blinding used (if applicable)? | Not reported (inferred that there was no blinding). |

Table S10. Data extraction details - Barzin et al., 2022

| Domain | Extraction Questions | Detailed Answers |
| --- | --- | --- |
| A. STUDY IDENTIFICATION | A1. First author and year of publication | Barzin et al., 2022 |
|  | A2. Study title | Predictive Factors of Cholelithiasis After Prophylactic Administration of Ursodeoxycholic Acid Following Laparoscopic Bariatric Surgery: Tehran Obesity Treatment Study |
|  | A3. Publishing journal | Obesity Surgery |
|  | A4. Country/region where conducted | Iran |
|  | A5. Language of publication | English |
|  | A6. Funding source | Department of Research and Technology of Shahid Beheshti University of Medical Sciences (dregi​strys​bmu.​ac.​ir). |
| B. METHODOLOGICAL CHARACTERISTICS | B1. What was the study design? | Prospective cohort study (part of the Tehran Obesity Treatment Study - TOTS). |
|  | B2. What was the data collection period? | March 2013 to March 2018. |
|  | B3. What was the follow-up duration? | 24 months postoperatively. |
|  | B4. Was the study multicentric? | Yes (three university hospitals in Tehran). |
|  | B5. What was the study setting? | University hospitals |
| C. STUDY POPULATION | C1. What was the total sample size? | 2629 patients included in the analysis. |
|  | C2. How many patients received UDCA? | All 2629 patients (it was a standard protocol of the TOTS study). |
|  | C3. What was the mean/median age of patients? | 38.8 ± 11.6 years. |
|  | C4. What was the percentage of female patients? | 79.7% (2049/2629). |
|  | C5. What was the mean/median preoperative BMI? | 44.7 ± 5.8 kg/m². |
|  | C6. What types of bariatric surgery were included? | Sleeve Gastrectomy (SG) and Gastric Bypass (GB - Roux-en-Y gastric bypass or One Anastomosis Gastric Bypass). |
| D. UDCA DOSING PROTOCOL | D1. What UDCA dosage was used? | 600 mg/day (300 mg x 2). |
|  | D2. Was the dosage based on body weight? | No. |
|  | D3. If so, what was the dosage per kg? | Not applicable. |
|  | D4. What was the daily administration frequency? | Twice daily. |
|  | D5. Was the dosage adjusted during treatment? | Not reported. |
|  | D6. If so, what were the criteria for adjustment? | Not applicable. |
| E. TREATMENT DURATION PROTOCOL | E1. What was the total duration of UDCA treatment? | 6 months. |
|  | E2. Was the duration predefined or criteria-based? | Predefined. |
|  | E3. If criteria-based, what were they? | Not applicable. |
|  | E4. Did any patients discontinue earlier than planned? | Yes (15 patients were excluded from the analysis). |
|  | E5. If so, what were the reasons for discontinuation? | Generalized rashes. |
|  | E6. What was the protocol adherence rate? | Not explicitly reported for adherence rate, but discontinuation due to AE was 15/2629 (0.57%). |
| F. INITIATION TIMING PROTOCOL | F1. When was UDCA initiated in relation to surgery? | Postoperatively. |
|  | F2. If preoperatively, how many days before surgery? | Not applicable. |
|  | F3. If postoperatively, how many days after surgery? | Not specified in exact days, but "routine 6-month prophylactic UDCA regimen after surgery". |
|  | F4. Was the initiation timing standardized for all patients? | Yes (all patients received the 6-month postoperative regimen). |
|  | F5. Were there specific criteria for determining initiation? | No (systematic prescription). |
|  | F6. If so, what criteria were used? | Not applicable. |
| G. MONITORING AND FOLLOW-UP | G1. Was monitoring performed during UDCA use? | Yes. |
|  | G2. If so, what was the monitoring frequency? | Every 3 months in the first year, and at 18 and 24 months postoperatively. |
|  | G3. What tests were used for monitoring? | Abdominal ultrasound (mainly). Biochemical tests (Fasting glucose, TG, Total Cholesterol, HDL, LDL) collected at each visit. |
|  | G4. How was gallstone formation assessed? | Abdominal ultrasound. |
|  | G5. What was the frequency of cholelithiasis assessment? | Every 3 months in the first year, and at 18 and 24 months postoperatively. |
|  | G6. Were there predefined criteria to discontinue UDCA? | Yes (in case of generalized rashes, 15 patients were excluded from the analysis). |
| H. EFFICACY OUTCOMES | H1. What was the incidence of cholelithiasis in the UDCA group? | 10.8% (283 of 2629 patients) at 24 months. (No control group without UDCA in this study). |
|  | H2. What was the incidence of cholelithiasis in the control group (if applicable)? | Not applicable (no control group without UDCA). |
|  | H3. What was the mean time to cholelithiasis development? | The highest incidence occurred in the first year. |
|  | H4. Was there a statistically significant difference? | Not applicable (no control group without UDCA for direct efficacy comparison). |
|  | H5. What was the calculated preventive efficacy? | Not applicable (only incidence in the UDCA group). |
|  | H6. How many patients required cholecystectomy? | Not reported. |
| I. SAFETY AND ADVERSE EVENTS | I1. Were UDCA-related adverse events reported? | Yes. |
|  | I2. If so, what were the main adverse events? | Generalized rashes. |
|  | I3. What was the incidence of adverse events? | 15 patients (approximately 0.57% of the total sample). |
|  | I4. Was there discontinuation due to adverse events? | Yes (15 patients). |
|  | I5. If so, what was the discontinuation rate? | 0.57%. |
|  | I6. Were contraindications to use reported? | Patients with previous cholecystectomy or preoperative asymptomatic or symptomatic cholelithiasis were excluded. |
| J. VARIATIONS BY SURGERY TYPE | J1. Did the protocol vary according to the type of bariatric surgery? | No, the UDCA protocol was the same for SG and GB. |
|  | J2. If so, what were the differences by surgery type? | Not applicable. |
|  | J3. Was there justification for protocol variations? | Not applicable. |
|  | J4. Which surgery type showed the greatest benefit? | There is no comparison of UDCA efficacy between surgery types in this study; only the incidence of cholelithiasis was similar between surgery types. |
|  | J5. Were specific recommendations reported per procedure? | Not applicable. |
| K. LIMITATIONS AND QUALITY | K1. What limitations were reported by the authors? | Difficulty of ultrasonographic assessment of the gallbladder in obese patients and possible inconsistency in ultrasound diagnosis; relatively short follow-up duration (2 years); non-randomized allocation and consecutive selection of surgeries (limitation of baseline imbalance); retrospective analysis of prospective data. |
|  | K2. Were conflicts of interest declared? | No ("The authors declare no conflicts of interest."). |
|  | K3. What was the loss to follow-up rate? | Not reported for the final sample of 2629 patients. (3218 patients recruited, 2629 included in the analysis, 15 excluded due to UDCA discontinuation). |
|  | K4. Did the study have adequate statistical power? | Not specified in the text, but it is a cohort study. |
|  | K5. Were appropriate randomization methods used (if RCT)? | Not applicable (cohort study). |
|  | K6. Was adequate blinding used (if applicable)? | Not applicable (cohort study). |

Table S11. Data extraction details - Haal et al., 2022

| Domain | Extraction Questions | Detailed Answers |
| --- | --- | --- |
| A. STUDY IDENTIFICATION | A1. First author and year of publication | Haal et al., 2022 |
|  | A2. Study title | Cost-effectiveness of ursodeoxycholic acid in preventing new-onset symptomatic gallstone disease after Roux-en-Y gastric bypass surgery |
|  | A3. Publishing journal | BJS |
|  | A4. Country/region where conducted | Not explicitly reported (referring to the UPGRADE trial, conducted in the Netherlands) |
|  | A5. Language of publication | English |
|  | A6. Funding source | The Netherlands Organization for Health Research and Development (ZonMw), Zambon Netherlands, Foundation for Clinical Research of the Slotervaart Hospital, Spaarne Gasthuis Academy, and Amsterdam Gastroenterology Endocrinology Metabolism. |
| B. METHODOLOGICAL CHARACTERISTICS | B1. What was the study design? | Economic evaluation (cost-effectiveness and cost-utility) alongside a multicenter Randomized Clinical Trial (RCT) (UPGRADE trial). |
|  | B2. What was the data collection period? | Not specified in the economic evaluation, but the UPGRADE RCT was from January 2017 to October 2018 for patient inclusion. |
|  | B3. What was the follow-up duration? | 2 years. |
|  | B4. Was the study multicentric? | Yes (UPGRADE trial was multicenter, with three high-volume bariatric centers). |
|  | B5. What was the study setting? | Hospital (bariatric centers). |
| C. STUDY POPULATION | C1. What was the total sample size? | 705 patients in the analyzed subpopulation (without pre-surgical gallstones, underwent RYGB). |
|  | C2. How many patients received UDCA? | 348 patients. |
|  | C3. What was the mean/median age of patients? | UDCA group: 44.9 (11.2) years; Placebo group: 44.1 (11.2) years. |
|  | C4. What was the percentage of female patients? | UDCA group: 76.7%; Placebo group: 75.9%. |
|  | C5. What was the mean/median preoperative BMI? | UDCA group: 39.8 (4.6) kg/m²; Placebo group: 40.0 (4.6) kg/m². |
|  | C6. What types of bariatric surgery were included? | Roux-en-Y Gastric Bypass (RYGB). (The original RCT included SG, but the economic analysis focused only on RYGB). |
| D. UDCA DOSING PROTOCOL | D1. What UDCA dosage was used? | 900 mg/day. |
|  | D2. Was the dosage based on body weight? | Not reported. |
|  | D3. If so, what was the dosage per kg? | Not applicable. |
|  | D4. What was the daily administration frequency? | Not specified (but generally 900 mg is a single dose or divided into 2-3 times daily). |
|  | D5. Was the dosage adjusted during treatment? | Not reported. |
|  | D6. If so, what were the criteria for adjustment? | Not applicable. |
| E. TREATMENT DURATION PROTOCOL | E1. What was the total duration of UDCA treatment? | 6 months. |
|  | E2. Was the duration predefined or criteria-based? | Predefined. |
|  | E3. If criteria-based, what were they? | Not applicable. |
|  | E4. Did any patients discontinue earlier than planned? | Yes (discussed in the clinical article of the UPGRADE trial, not specifically in the economic evaluation). |
|  | E5. If so, what were the reasons for discontinuation? | Not reported in the economic evaluation. |
|  | E6. What was the protocol adherence rate? | Not explicitly reported in the economic evaluation. |
| F. INITIATION TIMING PROTOCOL | F1. When was UDCA initiated in relation to surgery? | Postoperatively. |
|  | F2. If preoperatively, how many days before surgery? | Not applicable. |
|  | F3. If postoperatively, how many days after surgery? | Not specified in exact days, but "for 6 months". The UPGRADE protocol (Haal et al., 2020) mentions ideal initiation within 2 weeks, but up to 8 weeks. |
|  | F4. Was the initiation timing standardized for all patients? | Yes (standardized in the original RCT). |
|  | F5. Were there specific criteria for determining initiation? | No (systematic prescription in the RCT). |
|  | F6. If so, what criteria were used? | Not applicable. |
| G. MONITORING AND FOLLOW-UP | G1. Was monitoring performed during UDCA use? | Yes (as per original RCT). |
|  | G2. If so, what was the monitoring frequency? | Monitoring of symptomatic disease for 24 months. |
|  | G3. What tests were used for monitoring? | Not detailed in this article, but the RCT (UPGRADE trial) involved clinical follow-up and ultrasound. |
|  | G4. How was gallstone formation assessed? | Assessed as "symptomatic gallstone disease". |
|  | G5. What was the frequency of cholelithiasis assessment? | 24 months (primary endpoint of symptomatic disease). |
|  | G6. Were there predefined criteria to discontinue UDCA? | Not reported. |
| H. EFFICACY OUTCOMES | H1. What was the incidence of cholelithiasis in the UDCA group? | 3.4% (336 of 348 patients **remained free** of symptomatic disease, so 12/348 = 3.4% developed it). |
|  | H2. What was the incidence of cholelithiasis in the control group (if applicable)? | 9.2% (324 of 357 patients **remained free**, so 33/357 = 9.2% developed it). |
|  | H3. What was the mean time to cholelithiasis development? | Not reported. |
|  | H4. Was there a statistically significant difference? | Yes (Relative Risk 1.06; P = 0.002 for "remaining free of symptomatic disease"). |
|  | H5. What was the calculated preventive efficacy? | 63% reduction in the risk of developing symptomatic gallstone disease (9.2% vs 3.4%). |
|  | H6. How many patients required cholecystectomy? | The exact number of cholecystectomies not reported, but mentions "fewer cholecystectomies in the UDCA group". |
| I. SAFETY AND ADVERSE EVENTS | I1. Were UDCA-related adverse events reported? | Not detailed in this economic evaluation article, but the RCT article (UPGRADE trial) would address this. |
|  | I2. If so, what were the main adverse events? | Not applicable. |
|  | I3. What was the incidence of adverse events? | Not applicable. |
|  | I4. Was there discontinuation due to adverse events? | Not applicable. |
|  | I5. If so, what was the discontinuation rate? | Not applicable. |
|  | I6. Were contraindications to use reported? | Not applicable. |
| J. VARIATIONS BY SURGERY TYPE | J1. Did the protocol vary according to the type of bariatric surgery? | No, this analysis focused only on RYGB. |
|  | J2. If so, what were the differences by surgery type? | Not applicable. |
|  | J3. Was there justification for protocol variations? | Not applicable. |
|  | J4. Which surgery type showed the greatest benefit? | Not applicable. |
|  | J5. Were specific recommendations reported per procedure? | Efficacy was confirmed only for patients who underwent RYGB. |
| K. LIMITATIONS AND QUALITY | K1. What limitations were reported by the authors? | Hospital care volume data for patients from failed hospitals required imputation; data from some sources (outpatient consultations, diagnostic/therapeutic procedures from non-researched locations) were missing; data on hospital-prescribed medications and travel expenses were not collected; correction for baseline difference in health utility; 2-year time horizon (ideally lifetime for economic evaluation). |
|  | K2. Were conflicts of interest declared? | No ("The authors declare no conflict of interest"). |
|  | K3. What was the loss to follow-up rate? | Not explicitly specified for this subpopulation (705 patients). (985 patients initially included in the RCT, 959 in the modified intention-to-treat analysis). |
|  | K4. Did the study have adequate statistical power? | Not applicable (economic evaluation). |
|  | K5. Were appropriate randomization methods used (if RCT)? | Yes (data from an RCT, the UPGRADE trial). |
|  | K6. Was adequate blinding used (if applicable)? | Yes (original RCT was double-blind). |
